# Drought adaptation in spring wheat seedlings relies on coordinated deep root architecture and cortical tissue allocation

**DOI:** 10.3389/fpls.2026.1846481

**Published:** 2026-06-08

**Authors:** Xinying Li, Congcong Guo, Chengxin Bai, Shiji Wang, Xiaoya Gao, Jiahui Huo, Fuyang Cui, Hong Fan, Xiaoyuan Bao, Cai Zhao

**Affiliations:** 1 State Key Laboratory of Aridland Crop Science, College of Agronomy, Gansu Agricultural University, Lanzhou, China; 2 Seed Industry Research Institute of Gansu Provincial University, Lanzhou, China

**Keywords:** cortical allocation, deep root configuration, drought tolerance, high-throughput phenotyping platform, spring wheat

## Abstract

**Introduction:**

Root anatomical traits and spatial architecture play a critical role in crop water acquisition and utilization, directly impacting drought tolerance. However, comprehensive studies examining the synergistic effects of deep root configuration and cortical tissue organization under drought stress during the seedling stage remain scarce. Additionally, the underlying physiological mechanisms are not yet well understood.

**Methods:**

In this study, we utilized a high-throughput, paper-based phenotyping platform to simulate drought stress using 10% PEG. An efficient, multi-trait evaluation framework was employed to classify the 28 tested genotypes into five drought tolerance categories.

**Results:**

This approach enabled the identification of drought-tolerant cultivars “Ruichun 1,” “Ningchun 11,” and “Ningchun 57,” as well as the drought-sensitive cultivar “Dingxi 48.” Root traits, including maximum depth, convex hull area, and plant height, demonstrated strong explanatory power and could serve as valuable phenotypic indicators for seedling stage screening. Our findings suggest that drought adaptation in spring wheat involves a strategic coupling in which specific cortical configurations facilitate the development of deep root architecture. While previous studies have often focused on individual parameters, we show that drought-tolerant genotypes optimize root growth in deeper segments of the growth medium by adjusting cortical tissue proportions, potentially minimizing metabolic costs.

**Discussion:**

This integrated perspective offers a detailed physiological framework for understanding drought resilience and moves toward a mechanism-based interpretation of resource reallocation. However, it is important to note that these results were obtained using a paper-based phenotyping platform under PEG-induced osmotic stress, reflecting the genotypic potential at the seedling stage rather than actual field drought tolerance. In conclusion, combining the paper-based high-throughput phenotyping platform with a multi-trait evaluation framework allows for the accurate classification of drought tolerance types and the efficient identification of representative spring wheat cultivars. The findings emphasize the importance of deep root configuration and optimized cortical allocation as fundamental components of the root structural basis for drought adaptation in spring wheat. These results provide clear phenotypic targets for early-stage screening, which should be further validated at later developmental stages and under field conditions before being applied in breeding programs.

## 1 Introduction

Drought stress is a major abiotic factor that limits crop productivity worldwide (Vadez et al., 2024). Spring wheat (*Triticum aestivum* L.) is widely grown in arid and semi-arid regions, where frequent water deficits make drought stress a critical challenge for maintaining stable yields. Recent studies have identified significant genotypic variation in root responses to water limitation (Rahnama et al., 2024). Given this variation, further investigation into the mechanisms of drought tolerance in spring wheat roots is essential. Moreover, developing strategies to enhance drought tolerance in spring wheat is equally important.

The root system is essential for anchorage, water and nutrient absorption, and transport within plants. As the primary interface for water uptake and a key tissue for drought signal perception, it plays a central role in plant growth and yield formation (Holz et al., 2024). Root system architecture (RSA) is considered a potential “second Green Revolution” for crop improvement, exhibiting significant phenotypic and genetic diversity (Rahnama et al., 2011; Gifford et al., 2024). Under drought stress, accurately characterizing root growth patterns and spatial distribution is crucial for optimizing RSA. Studies indicate that optimizing RSA traits through phenotypic selection and breeding programs enhances water and nutrient acquisition in resource-limited environments, thereby supporting aboveground functions. This, in turn, improves drought tolerance and reduces yield losses (Rahnama et al., 2019, 2024).

To enhance crop drought tolerance, it is essential to understand the synergistic relationships among various root adaptive traits. Both rice and wheat are monocotyledonous cereals with conserved root anatomical features, making insights from rice into drought-adaptive root traits valuable for improving drought tolerance in wheat. In rice, the strategic coordination between root length and structural architecture facilitates deep root deployment (Uga et al., 2013; Ouyang et al., 2020). This characteristic improves drought tolerance by optimizing resource allocation for downward root expansion into deeper zones of the growth medium, thereby supporting sustainable water acquisition during periods of water deficit. An optimal root anatomical organization under drought stress is characterized by specific tissue proportions, including lower cortical/stele area ratios, lower xylem/stele area ratios, and higher aerenchyma/cortex area ratios (Lynch et al., 2021). Deep rooting plays a pivotal role in enhancing crop drought tolerance. The combination of reduced cortical/stele area ratios and increased aerenchyma/cortex area ratios may lower metabolic costs, as reduced cortical tissue and increased aerenchyma formation have been shown to decrease root respiration and carbon costs in previous studies (Lynch et al., 2021; Yamauchi et al., 2021). Furthermore, studies have shown that wheat cultivars with smaller xylem areas exhibit greater drought tolerance compared to those with larger xylem areas. This further underscores the adaptive significance of a reduced xylem/stele area ratio under drought conditions (Li et al., 2021). Collectively, adjusting root tissue proportions is closely linked to crop drought tolerance and represents a crucial step in elucidating drought adaptation mechanisms and guiding drought-tolerant breeding strategies.

Selecting a high-throughput analytical approach to evaluate root growth is crucial, as root system architecture (RSA) is closely linked to root function and provides valuable insights for crop improvement. RSA analyses must be simple and efficient to effectively support experimental crop improvement programs (Gifford et al., 2024). Traditional root analysis methods face several limitations, including labor-intensive ink staining procedures and excessive time requirements (Atkinson et al., 2019). For example, conventional greenhouse-based root phenotyping approaches are inefficient and low in throughput, making them unsuitable for large-scale quantitative RSA studies (Gill et al., 2022). Given these challenges, there is a pressing need for high-throughput screening methods to validate the effectiveness of specific root traits in defining ideal RSA ideotypes. These methods should ultimately integrate such traits into selection criteria for crop improvement. Various artificial cultivation systems, such as hydroponic culture and germination paper-based systems, are currently widely used in RSA research. Among these, the germination paper system was initially developed to study root angle in common bean and has since been applied to root studies in maize (Le Marie et al., 2014), cotton (Atkinson et al., 2019), wheat (Rahnama et al., 2011; Adeleke et al., 2020), barley, and oilseed rape (Nagel et al., 2012). These studies have provided crucial evidence for identifying key RSA traits and cultivar development. The high heritability of relevant traits and the ease of environmental control in artificial cultivation systems have established a strong foundation for RSA and root anatomy studies. In particular, paper-based systems enable precise regulation of stress conditions using controlled nutrient or osmotic solutions. This facilitates stable drought simulation, reduces environmental variability, and allows for accurate assessments of trait heritability. Consequently, high-throughput phenotyping approaches can robustly evaluate root traits related to drought tolerance.

In summary, existing studies still have limitations in the comprehensive evaluation of drought tolerance in wheat. Most previous research on seedling-stage drought tolerance has primarily focused on germination vigor, aboveground growth traits, physiological indicators, or basic root architecture measurements. Specifically, these studies have inadequately addressed the combined contributions of whole-root architecture, stratified root anatomical traits, and aboveground traits to drought tolerance mechanisms. This gap limits the accuracy and interpretability of drought-tolerant germplasm screening. To address these shortcomings, the present study systematically integrates root system architecture traits, root anatomical traits from different root segments, and aboveground traits into a unified drought tolerance evaluation framework, thereby enhancing the scientific rigor and reliability of the screening process. The novelty of this study lies not in the statistical methods themselves-which are well-established and widely used in crop drought tolerance evaluation-but in the selection and combination of traits incorporated into the multi-trait evaluation framework. Accordingly, this study aims to answer three key biological questions: (1) Using a multi-trait comprehensive evaluation framework, we aim to identify drought-tolerant cultivars and determine which root and aboveground phenotypic traits most significantly contribute to drought tolerance in spring wheat at the seedling stage; (2) How root architectural and anatomical traits interact with aboveground drought-related traits to shape drought tolerance; and (3) Whether and how deep root configuration contributes to enhanced drought tolerance through coordinated root structural and anatomical adaptation. To achieve these objectives, we employed a high-throughput paper-based phenotyping platform combined with a multi-trait evaluation framework. The outcomes of this study are expected to provide a quantitative phenotypic basis for characterizing drought-adaptive traits and their associations in spring wheat seedlings under controlled osmotic stress. Given the inherent limitations of PEG-induced stress in simulating field drought, these findings should be regarded as a preliminary screening framework for assessing genotypic potential. After further validation under field conditions across multiple developmental stages, these results may inform future breeding strategies for drought-resilient spring wheat germplasm.

## 2 Materials and methods

### 2.1 Plant materials

In this study, 28 spring wheat cultivars recently approved at the national or provincial level were used as experimental materials. These cultivars were collected from arid and semi-arid ecological regions of northwestern China, including Ningxia, Inner Mongolia, Gansu, Xinjiang, and Qinghai. Previous studies have shown that they exhibit substantial variation in drought tolerance at the seedling stage. All plant materials were provided by the Wheat Research Group, Department of Plant Breeding and Genetics, Gansu Agricultural University. Detailed cultivar information is provided in **Supplementary Table S1**.

### 2.2 Experimental design

#### 2.2.1 Controlled-environment seedling experiment

The experiment was conducted from December 2, 2024, to April 13, 2025, in an artificial climate chamber at the State Key Laboratory of Aridland Crop Science, Gansu Agricultural University (36°03′N, 103°44′E). Spring wheat seeds were surface-sterilized with 75% ethanol for 2 min, followed by 5% sodium hypochlorite for 8 min, and then rinsed 5–8 times with sterile distilled water. The sterilized seeds were placed in Petri dishes measuring 15 cm in diameter and 1.5 cm in height, lined with filter paper. Each dish contained 60 healthy, uniform, and undamaged seeds. The seeds were germinated in darkness for 3 days at a day/night temperature of 25/22 °C. After germination, seedlings with uniform vigor and balanced root and shoot growth were selected from each dish and transferred to a paper-based phenotyping platform. Seedling uniformity was verified by measuring root and shoot lengths with a ruler, and seedlings with values within ±5% of the dish mean were selected. For each cultivar, 30 paper pouches were prepared, with one seedling placed at the center of each filter paper to ensure close root-paper contact. A 1/4-strength Hoagland nutrient solution was added to support seedling growth. The pouches were randomly arranged within the growth chamber to minimize positional effects. In total, 840 paper pouches were used. At 24 h after transplanting, 15 of the 30 pouches for each cultivar were randomly assigned to the drought stress treatment, whereas the remaining 15 served as controls and continued to receive 1/4-strength Hoagland nutrient solution.

When the plants reached the two-leaf stage, a 7-day water stress treatment was imposed. Two treatments were established: normal water supply as the control (CK, 0% PEG 6000) and drought stress (DS, 10% PEG 6000), each with five replicates. The 10% PEG 6000 solution, with an osmotic potential of approximately -0.15 MPa, was used to impose moderate osmotic stress at the seedling stage. This concentration was selected to induce adaptive responses and distinguish genotypic differences in drought tolerance without causing complete growth cessation (Ding et al., 2016). The nutrient solution was adjusted to pH 6.0 ± 0.1 and replaced every 3 days. Environmental conditions were maintained as follows: day/night temperature, 28 ± 2/25 ± 2 °C; photoperiod, 14/10 h; relative humidity, 40-50%; and light intensity, 600 µmol m^-2^ s^-1^. During the experiment, aeration was supplied twice daily for 30 min each time.

#### 2.2.2 Field experiment for yield evaluation

The 2025 field experiment was conducted at the Jingtai County Experimental Station in Gansu, China (104°15′E, 37°14′N). Spring wheat germplasm was grown under two treatments: non-stress (NS) and drought stress (DS), with three replicates per treatment in a completely randomized block design. Each plot measured 2.52 m × 1.4 m, with 18 cm row spacing and a seeding rate of 515.87 kg·ha^-1^. Both treatments were drip-irrigated, and the monthly rainfall and irrigation data are summarized in **Supplementary Table S2**. The DS plots received approximately 50% of the irrigation provided to the NS plots during key growth stages. Grain yield per plot (GYP) was determined by harvesting all plants and recording the total grain weight. This data was subsequently used for drought-index and trait correlation analyses.

### 2.3 Determination of the above-ground traits

**Figure 1:**
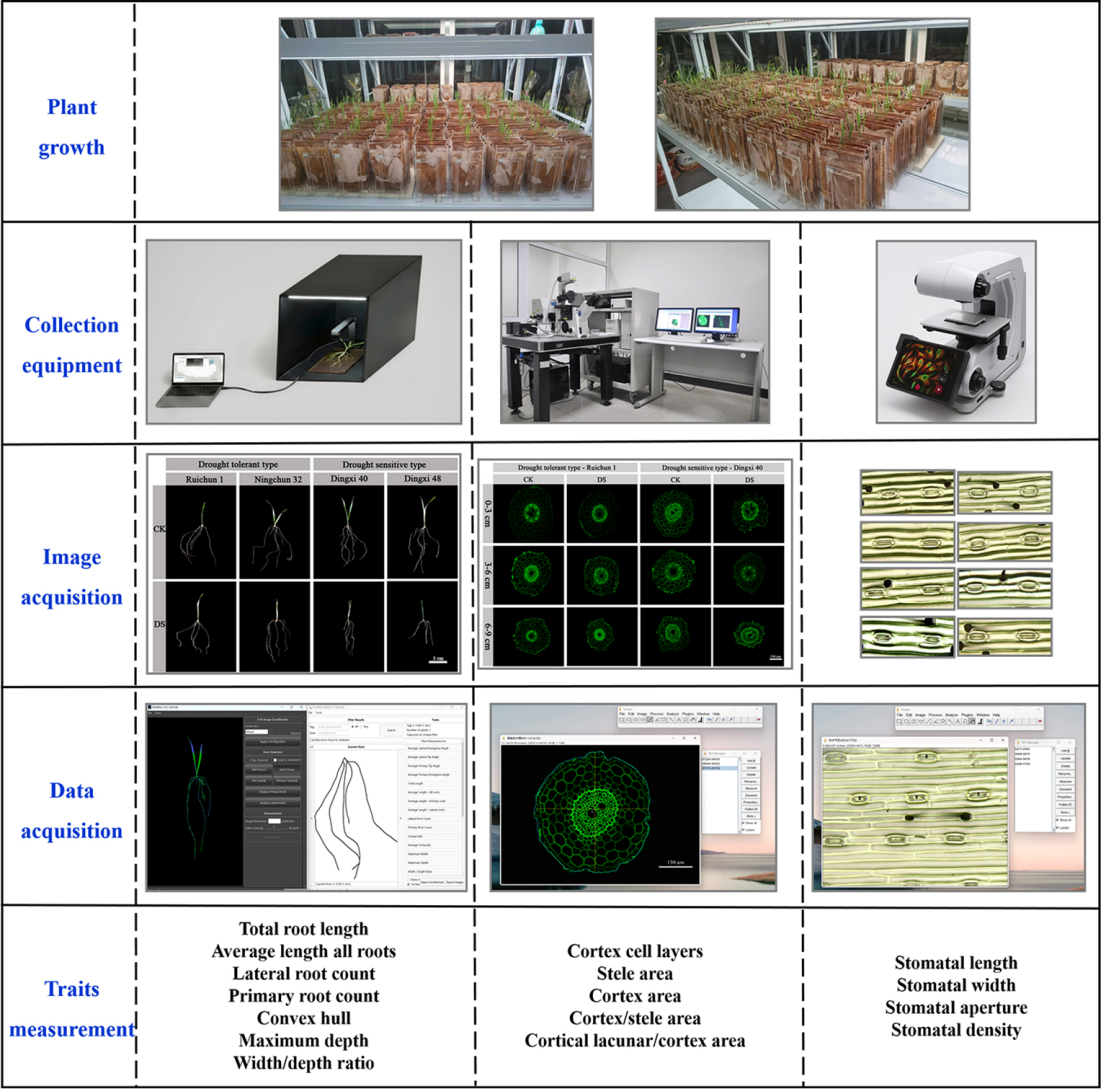
A flow diagram for investigating wheat root architecture, root cross-sections, and leaf stomata.

Sample collection was conducted on the 7th day of drought stress treatment. For each treatment, three wheat plants were randomly selected for the determination of morphological traits. Plant height from coleoptile node to main stem apex was measured with a ruler. Leaf area was calculated using the length-width coefficient method (Ma et al., 2022): LA = L × W × 0.75, where L is leaf length and W is leaf width. On the 7th day after drought stress treatment, leaf stomatal characteristics were measured between 10:30 and 12:00 following the method described by Wang et al (Wang and Chang, 2024). Briefly, stomatal traits were assessed using a combined nail polish and transparent tape method, with measurements conducted on the second fully expanded leaf from the top (Pathoumthong et al., 2023). Nail polish was evenly applied to the abaxial leaf surface and allowed to air-dry for 2–5 min. Transparent tape was then placed over the dried nail polish layer to obtain an impression, which was subsequently mounted for observation. Stomatal images were captured using an optical microscope (LEICA 2500, Germany). For each image, five fields of view were randomly selected, and within each field, five stomata were randomly chosen for analysis. ImageJ software was used to determine stomatal length (µm), stomatal width (µm), and stomatal aperture (µm) (**Figure 1**). The area of each field was recorded. Stomatal number per image was counted and divided by the area to calculate mean stomatal density (stomata mm^-2^) per field.

### 2.4 Determination of the RSA

The imaging-germination platform included plant germination pouches, a pouch holder, a small photographic lightbox, a high-resolution scanner, and a laptop computer (**Figure 1**). The paper-based system utilized Whatman No.1 filter paper for the germination pouches, which were standard germination bags measuring 180 mm × 125 mm. The pouch holder was a rectangular structure (20 × 15 × 30 cm) made of ten transparent acrylic panels, designed to hold nine germination pouches simultaneously, ensuring uniform placement and management of seedlings.

The germination pouch cabinet was also rectangular (200 × 100 × 50 cm) with four internal shelves (100 × 50 cm each). An adjustable 220 V LED light strip was installed above each shelf to provide supplemental illumination, ensuring precise control of the photoperiod during cultivation. The small photographic lightbox, measuring approximately 60 cm × 60 cm × 60 cm, was lined internally with non-reflective black materials (e.g., black foam board or fabric) to minimize reflections and shadows during image acquisition. Evenly distributed LED strips inside the lightbox ensured uniform illumination across the imaging area, providing a stable background and lighting environment that improved image clarity and consistency.

Root samples were collected on the 7th day of drought stress treatment. Three representative wheat plants were randomly selected from each treatment group. Roots were kept hydrated and immediately scanned (without air-drying) to prevent shrinkage. Prior to scanning, roots were gently separated using soft forceps in a water-filled tray to reduce overlap while preserving their natural architecture. Whole root systems were scanned at 1200 dpi using a high-resolution scanner (GP1600AF, COMET, China). The imaging system was controlled by a laptop computer (LAPTOP-5MNUD00G, HONOR, China) to maintain stable and uniform lighting conditions during scanning, optimizing image quality. The acquired digital images were saved in TIFF format. After image acquisition, preprocessing was performed using Photoshop (Ps). This included scaling and cropping to standardize image size, mild blurring to reduce background noise and improve feature recognition, and horizontal flipping to ensure consistent orientation across samples. These steps enhanced image clarity and facilitated subsequent batch analysis. To resolve any remaining overlaps, distinguishable roots were manually separated using Photoshop’s selection tools. Skeleton-tracking algorithms in RootNav were then employed to automatically trace root paths. The preprocessed images were imported into RootNav and RhizoVision Explorer software for the quantitative extraction of root traits (specific parameters are listed in **Table 1**). To ensure standardized data management, all images were systematically renamed according to predefined sample identification codes.

**Table 1:** Root system architecture traits summary.

No.	Parameter	Unit
1	Plant height	cm
2	Leaf area	cm
3	Stomatal pore length	μm
4	Stomatal pore width	μm
5	Stomatal aperture	–
6	Stomatal density	stomata mm^-2^
7	Total root length	mm
8	Average length of all roots	mm
9	Lateral root count	count
10	Primary root count	count
11	Convex hull area	mm^2^
12	Maximum root depth	mm
13	Width/depth ratio	–
14	Cortex cell layers	–
15	Stele area	μm^2^
16	Cortex area	μm^2^
17	Porosity area	μm^2^
18	Cortex/stele area	–
19	Lacunar/cortex area	–
20	Network area	mm^2^
21	Root volume	mm^3^
22	Average diameter	mm
23	Number of root tips	count

### 2.5 Determination of the root anatomical structure

Samples were collected on the 7th day of drought stress treatment. Three 0.5-cm root segments were excised from the primary seminal root of each plant at positions 0–3 cm, 3–6 cm, and 6–9 cm from the root tip. These sampling positions were selected to capture a longitudinal anatomical gradient along the primary seminal root, from the distal region near the root apex to the more differentiated regions closer to the root base. This design allowed us to examine spatial variation in cortical and stele-related anatomical traits along the root axis (Guo et al., 2025). Three biological replicates were used for each treatment. To minimize bias during anatomical analysis, all samples were coded prior to imaging, and the operator was blinded to both genotype and treatment during image acquisition and trait measurement. The collected root segments were initially preserved in 75% ethanol for anatomical analysis of root cortical tissues. Cortical tissue development was analyzed using acridine orange staining (Henry and Deacon, 1981). The stained root segments were then embedded in Tissue-Tek CRYO-OCT compound (Thermo Fisher Scientific, USA), rapidly frozen in liquid nitrogen, and stored at -20 °C. Transverse root sections with a thickness of 8 µm were prepared using a cryostat microtome (Leica CM1950, Germany) and immediately mounted onto microscope slides for imaging. Cross-sectional images were acquired using an inverted fluorescence microscope (Nikon, ECLIPSE Ti2-U, Japan) and a laser confocal microscope (FV-1000, Olympus, Japan) (**Figure 1**). Phenotypic analysis was performed using ImageJ software (NIH, USA). The extracted anatomical traits included cortex cell layers, stele area, cortex area, porosity area, cortex/stele area ratio, and lacunar/cortex area ratio.

### 2.6 Drought tolerance evaluation

A multi-trait comprehensive evaluation framework, incorporating the drought tolerance coefficient, membership function analysis, and a comprehensive drought tolerance evaluation value (D value), was employed, and the calculation methods for each component are described as follows:

The drought tolerance coefficient is expressed as the relative value between the stress treatment and the control, and is calculated using the following formula:

(1) $$\text{Drought tolerance coefficient}=\frac{{\text{X}}_{\text{i}}(\text{k})}{{\text{CX}}_{\text{i}}(\text{k})}$$

(2) $$\text{Coefficient of variation }(\%)=\frac{\text{SD}}{\bar{X}}$$

Principal component analysis was performed on the drought tolerance coefficients of all traits, followed by calculation of their corresponding membership function values μ(xj):

(3) $$\mu \left({\text{x}}_{\text{j}}\right)=\frac{\left({\text{x}}_{\text{j}}–{\text{x}}_{\min}\right)}{\left({\text{x}}_{\max}-{\text{x}}_{\min}\right)}\text{ j}=1,2,\cdots ,\text{n}$$

The weight of each comprehensive index (w_j) was calculated using the following formula:

(4) $${\text{w}}_{\text{j}}=\frac{{\text{p}}_{\text{j}}}{\sum_{\text{j}=1}^{\text{n}}{\text{p}}_{\text{j}}}\text{ j}=1,2,\cdots ,\text{n}$$

The D value serves as an integrated index for evaluating drought tolerance; a higher D_j value indicates stronger overall drought tolerance of the genotype. The D value was calculated using the following formula, a schematic diagram summarizing the stepwise integration of root and shoot traits into the D value is provided in **Supplementary Figure S1**:

(5) $$\text{D}=\sum_{\text{j}=1}^{\text{n}}\left[\text{u}({\text{x}}_{\text{j}})\times {\text{w}}_{\text{j}}\right]\text{ j}=1,2,\cdots ,\text{n}$$

To comprehensively account for root system characteristics, root traits were normalized and combined with the relative drought tolerance coefficients of each trait to assess drought tolerance.

(6) $${\text{Xi}}^{\prime }\left(\text{k}\right)=\frac{\left[{\text{X}}_{\text{i}}\left(\text{K}\right)-{\text{X}}_{\min}\right]}{\left[{\text{X}}_{\max}-{\text{X}}_{\min}\right]}$$

Where *Xi* (*k*) and C*Xi* (*k*) represent the measured values under drought stress treatment and normal irrigation treatment, respectively; SD denotes the standard deviation; $\bar{X}$ is the mean value of a given trait; x_j_ is the (i)th comprehensive index; x_min_ and x_max_ are the minimum and maximum values of the (i)th comprehensive index, respectively; w_j_ indicates the relative importance (weight) of the (i)th comprehensive index among all indices; p_j_ represents the contribution rate of the (i)th comprehensive index for each cultivar; and the *D* value is the *D* value of drought tolerance for each cultivar. Based on the *D* values, the drought tolerance of the tested cultivars can be classified. ${\text{Xi}}^{\prime }\left(\text{k}\right)$ denotes the normalized value of the data.

### 2.7 Statistical analysis

Microsoft Excel 2019 was used for data recording, organization, calculation of functions, and computation of D values. SPSS 27.0 (IBM, Armonk, NY, USA) was employed for analysis of variance, principal component analysis, correlation analysis, and data standardization. Microsoft Excel 2019 was also used to calculate membership function values and D values. Origin 2022b (OriginLab, Northampton, MA, USA) was applied for data visualization and hierarchical cluster analysis. Data are presented as means ± standard error (mean ± SE). One-way analysis of variance followed by the least significant difference (LSD) test was used to determine significant differences at the 95% or 99% confidence levels. Adobe Illustrator 2020 was used for figure assembly. Systematic clustering of Dc values was performed using the Euclidean distance method combined with hierarchical clustering.

## 3 Results

### 3.1 Drought tolerance coefficients and phenotypic trait analysis

**Figure 2:**
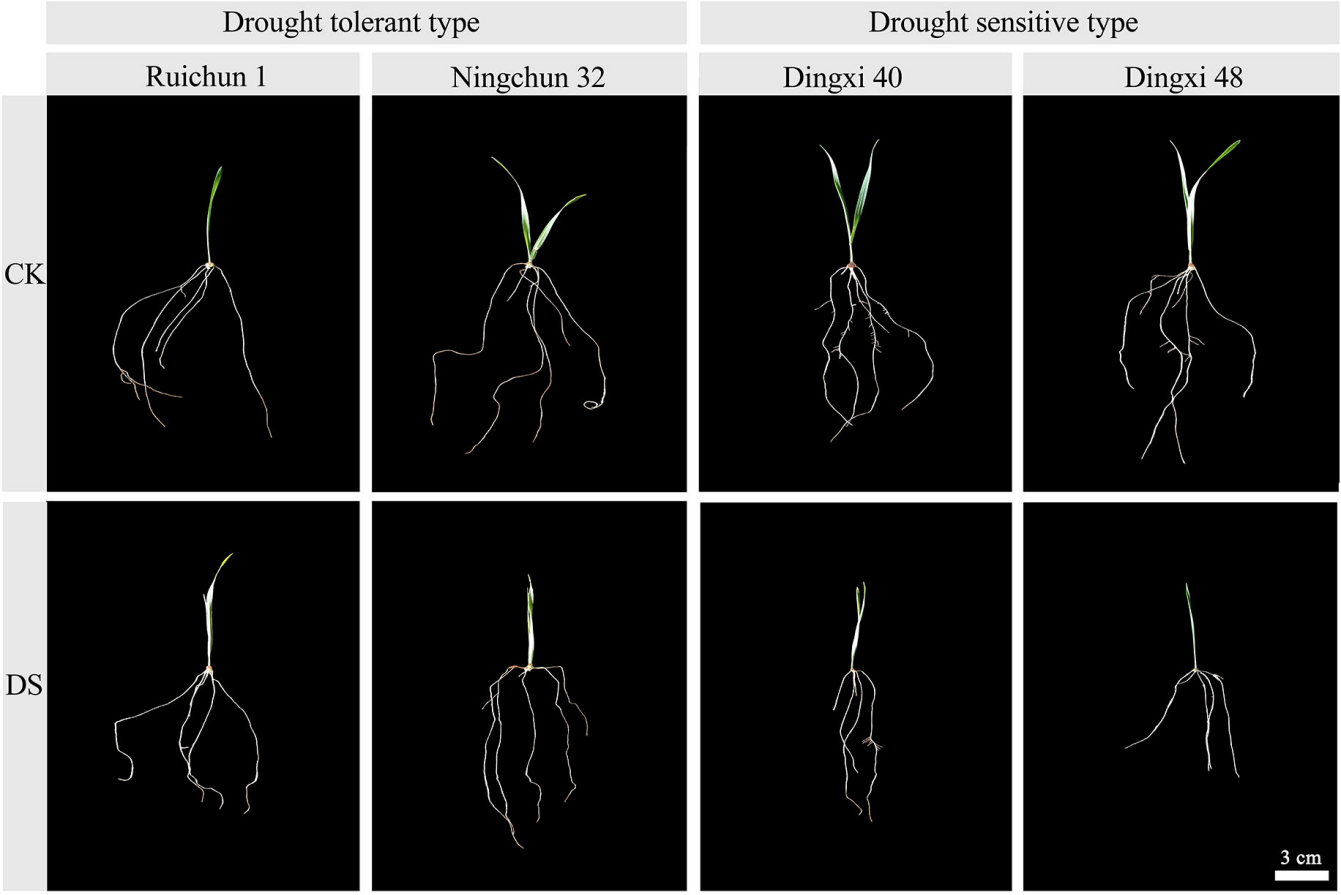
Growth performance of four spring wheat cultivars under control and drought stress conditions. CK, Control; DS, Drought stress.

**Figure 3:**
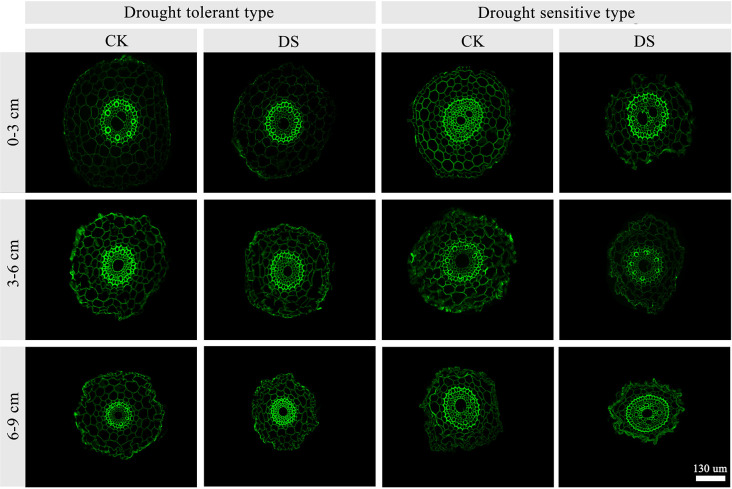
Root anatomical characteristics at different depths in two wheat cultivars under control and drought stress conditions. Root cross-sections of drought-tolerant and drought-sensitive types were observed at three depths (0–3 cm, 3–6 cm, and 6-9cm) under control and drought stress conditions. CK, Control; DS, Drought stress.

In this study, the drought tolerance coefficient, coefficient of variation, and normalized root trait values were calculated using Equations 1, 2, and 6, respectively. Drought stress significantly inhibited aboveground phenotypic traits of spring wheat (**Table 2**; **Figure 2**). The drought tolerance coefficients for aboveground traits ranged from 0.233 to 1.371. Among these, the coefficients for plant height, leaf area, stomatal length, stomatal width, and stomatal aperture were all below 1, indicating that these traits were generally suppressed to varying extents under drought conditions. In contrast, drought stress induced substantial variability in root system architecture among the 28 spring wheat cultivars, with trends distinct from those observed for aboveground traits (**Table 2**; **Figures 2**, **3**). Specifically, drought stress reduced total root length, average root length, number of lateral roots, number of primary roots, convex hull area, and maximum root depth, while increasing the width/depth ratio, number of cortical layers, cortical/stele area, and aerenchyma/cortical area. The increased width/depth ratio suggests a drought-induced adjustment in root system architecture, with more lateral than vertical root expansion. Meanwhile, the enlarged aerenchyma area may reduce living cortical tissue, thereby lowering root metabolic costs and supporting root growth and resource acquisition under water deficit. The coefficients of variation for total root length, average root length, number of lateral roots, number of primary roots, convex hull area, maximum root depth, width/depth ratio, number of cortical layers, cortical/stele area, and aerenchyma/cortical area were 27.674%, 30.376%, 35.304%, 16.060%, 39.813%, 9.457%, 30.683%, 27.745%, 35.779%, and 61.326%, respectively. These results indicate that, compared to aboveground traits, root architectural and anatomical traits exhibit greater variability and differentiation potential in response to drought stress. As a result, root-related traits may offer a broader selection space and serve as more discriminative phenotypic indicators for drought tolerance breeding. In particular, the high coefficients of variation for traits such as convex hull area, aerenchyma/cortex area, and lateral root count highlight their potential as key selection targets in breeding programs aimed at enhancing drought tolerance in spring wheat. Furthermore, the drought tolerance coefficients for grain yield per plot ranged from 0.403 to 1.020, with a coefficient of variation of 21.82%. This indicates that grain yield per plot varied among cultivars and was generally suppressed under drought condition (**Supplementary Table S4**).

**Table 2:** Drought tolerance coefficients of 28 spring wheat varieties under drought stress treatment.

Variety	PH	LA	SL	SW	PO	SD	TL	ALAR	LRC	PRC	CH	MD	W/DR	CL	CVA	CPA
Ruichun1	0.954	0.954	0.552	0.475	0.431	1.371	1.233	1.325	0.636	1.375	1.132	1.093	0.960	1.000	0.521	1.390
1538	0.734	0.734	0.918	0.932	0.937	0.706	0.605	0.617	1.000	0.800	0.683	0.922	0.795	1.000	1.326	0.370
2038	0.761	0.761	0.885	0.920	0.909	0.857	0.706	0.779	1.083	1.167	0.725	0.940	1.000	0.800	1.459	1.680
Ningchun 11	0.882	0.882	0.832	0.864	0.848	0.808	0.915	1.109	1.375	1.000	1.653	1.034	1.190	2.000	1.249	0.120
9396	0.908	0.908	0.761	0.722	0.684	1.200	0.811	0.923	0.938	1.000	0.669	0.932	0.764	1.250	0.740	2.710
Longchun 30	0.932	0.932	0.661	0.627	0.569	0.950	1.021	1.130	0.944	1.000	0.470	0.821	0.824	1.200	0.940	2.550
Longchun 34	0.906	0.906	0.761	0.734	0.700	1.087	1.229	1.188	0.600	1.000	0.846	0.868	1.234	0.667	0.780	2.320
Linmai 33	0.871	0.871	0.852	0.870	0.912	0.955	0.758	0.896	0.600	1.333	0.593	0.884	0.708	1.200	0.781	0.620
Ningchun 16	0.880	0.880	0.839	0.865	0.897	0.929	0.640	0.658	0.862	1.000	1.014	0.966	1.520	0.571	1.176	2.920
Ningchun 4	0.921	0.921	0.726	0.684	0.648	0.989	0.561	0.557	1.091	0.750	1.253	0.956	1.816	1.250	0.742	1.490
SM14	0.679	0.679	0.930	0.942	0.958	0.667	0.515	0.361	0.857	0.750	1.025	0.858	1.918	1.000	1.800	0.860
Ningchun 57	0.942	0.942	0.650	0.536	0.557	1.040	1.051	1.185	0.684	0.857	0.922	1.047	0.836	1.000	0.792	2.020
Ningchun 15	0.710	0.710	0.924	0.939	0.944	0.852	0.684	0.687	0.500	0.875	0.481	0.899	0.851	0.833	1.572	1.250
Ningchun 52	0.922	0.922	0.670	0.654	0.580	1.060	0.716	0.802	0.846	0.889	1.016	1.032	1.060	1.200	0.781	1.130
Bamai 19	0.926	0.926	0.666	0.647	0.572	0.933	0.662	0.676	1.846	1.333	0.476	0.753	1.294	0.667	1.098	0.960
Longchun 41	0.830	0.830	0.878	0.912	0.903	0.826	0.595	0.600	1.286	0.857	0.776	0.907	1.205	1.000	1.651	1.130
L623	0.847	0.847	0.866	0.925	0.981	0.810	0.618	0.650	1.000	1.000	0.552	0.818	1.067	1.000	1.240	0.670
L622	0.742	0.742	0.917	0.927	0.912	0.900	0.698	0.702	0.778	1.000	0.649	0.887	0.924	0.800	1.229	0.950
Yongliang 15	0.940	0.940	0.659	0.588	0.564	0.959	0.991	1.118	0.889	0.875	0.565	0.830	0.974	1.000	0.922	2.760
Dingxi 49	0.903	0.903	0.780	0.770	0.782	0.943	0.649	0.670	1.143	1.000	0.622	0.869	1.397	1.500	1.061	0.620
Dingxi 48	0.616	0.616	0.964	0.972	0.979	0.644	0.600	0.607	0.909	1.000	0.411	0.774	0.900	1.250	1.912	0.780
Dingxi 40	0.648	0.648	0.931	0.956	0.975	0.720	0.544	0.541	0.833	1.000	0.644	0.846	1.203	0.833	1.461	0.30
Lingxia 35	0.956	0.956	0.527	0.338	0.401	1.250	0.833	1.045	1.000	0.857	0.488	0.847	0.905	0.833	0.600	1.890
Bamai 20	0.977	0.977	0.493	0.233	0.249	1.368	0.741	0.888	1.800	1.000	0.427	0.856	0.667	1.000	0.557	2.750
Longchun 35	0.904	0.904	0.774	0.757	0.755	1.135	1.329	1.360	0.750	1.125	0.439	0.844	0.847	1.000	0.841	2.920
1407	0.884	0.884	0.825	0.834	0.835	1.043	0.840	1.060	0.350	0.900	0.797	1.021	0.707	0.833	0.928	1.050
Ningchun 32	0.893	0.893	0.810	0.824	0.816	0.963	0.725	0.887	0.909	1.000	1.274	0.978	1.453	1.200	0.857	0.720
Yong 2563	0.947	0.947	0.647	0.520	0.504	1.209	0.829	0.993	0.909	1.000	0.807	0.981	0.750	0.867	0.728	0.930
Mean	0.858	0.660	0.775	0.749	0.743	0.971	0.789	0.858	0.944	0.991	0.765	0.909	1.063	1.027	1.062	1.424
Max	0.977	0.960	0.964	0.972	0.981	1.371	1.329	1.360	1.846	1.375	1.653	1.093	1.918	2.000	1.912	2.920
Min	0.616	0.233	0.493	0.233	0.249	0.644	0.515	0.361	0.350	0.750	0.411	0.753	0.667	0.571	0.521	0.120
SD	0.102	0.220	0.132	0.197	0.205	0.193	0.218	0.261	0.333	0.159	0.304	0.086	0.326	0.285	0.380	0.873
CV(%)	11.891	33.333	17.036	26.280	27.562	19.906	27.674	30.376	35.304	16.060	39.813	9.457	30.683	27.745	35.779	61.326

PH, Plant height; LA, Leaf area; SL, Stomatal pore length; SW, Stomatal pore width; PO, Stomatal aperture; SD, Stomatal density; TL, Total Length; ALAR, Average Length of All roots; LRC, Lateral Root Count; PRC, Primary Root Count; CH, Convex hull area; MD, Maximum root depth; W/DR, Width/Depth Ratio; CL, Cortex cell layers; CVA, Cortex/Stele area; CPA, Lacunar/Cortex area.

### 3.2 Evaluation and screening of drought-tolerant cultivars

Under 10% PEG-induced drought stress, drought tolerance coefficients for 16 evaluation traits were calculated for 28 spring wheat cultivars. Principal component analysis (PCA), combined with the membership function method, was conducted. Based on the Kaiser criterion (eigenvalue > 1), the first four principal components (CI1-CI4) were retained. These components had eigenvalues greater than 1 and explained 82.369% of the total variance, while the remaining components contributed negligibly to the overall variation. The contribution rates of CI1-CI4 were 48.147%, 14.265%, 12.361%, and 7.596%, respectively (**Tables 3**, **4**).

**Table 3:** Coefficients and contribution proportions of comprehensive indices (CIx) for 28 spring wheat varieties under drought stress.

Principal factor	CI1	CI2	CI3	CI4
Factor weight	0.585	0.173	0.150	0.092
Characteristic value	7.704	2.282	1.978	1.215
Contribution rate	48.147	14.265	12.361	7.596
Cumulative contribution rate	48.147	62.412	74.773	82.369
Plant height	0.905	0.177	0.027	0.112
Leaf area	0.974	0.035	0.019	0.108
Stomatal length	-0.971	0.022	0.100	-0.093
Stomatal width	-0.952	0.096	0.114	-0.061
Stomatal aperture	-0.959	0.059	0.117	-0.071
Stomatal density	0.883	-0.038	0.191	0.211
Total root length	0.521	0.021	0.536	0.491
Average length of all roots	0.605	0.106	0.491	0.537
Lateral root count	0.239	0.063	-0.89	-0.037
Primary root count	0.086	0.052	-0.117	0.765
Convex hull area	0.061	0.862	0.213	-0.357
Maximum root depth	0.238	0.656	0.532	-0.151
Width/depth ratio	-0.17	0.294	-0.216	-0.686
Cortex cell layers	-0.042	0.734	-0.243	0.176
Cortex/stele area	-0.872	-0.116	-0.192	-0.188
Lacunar/cortex area	0.589	-0.492	0.271	-0.046

**Table 4:** Membership function values of each variety under drought stress.

U(Xi)	U(X1)	U(X2)	U(X3)	U(X4)
Ruichun1	1.000	0.635	0.937	1.000
1538	0.266	0.510	0.396	0.145
2038	0.639	0.671	0.415	0.000
Ningchun 11	0.239	0.948	0.648	0.275
9396	0.367	0.459	0.859	0.414
Longchun 30	0.296	0.645	0.900	0.609
Longchun 34	0.713	0.834	0.865	0.542
Linmai 33	0.427	0.744	0.613	0.572
Ningchun 16	0.762	0.494	0.711	0.012
Ningchun 4	0.293	0.238	0.841	0.434
SM14	0.156	0.345	0.199	0.011
Ningchun 57	0.592	0.536	1.000	0.723
Ningchun 15	0.288	0.397	0.317	0.214
Ningchun 52	0.439	0.262	0.848	0.660
Bamai 19	0.732	0.586	0.700	0.339
Longchun 41	0.329	0.474	0.509	0.006
L623	0.275	0.565	0.533	0.245
L622	0.471	0.553	0.421	0.217
Yongliang 15	0.374	0.551	0.945	0.591
Dingxi 49	0.021	0.497	0.678	0.478
Dingxi 48	0.000	0.510	0.000	0.040
Dingxi 40	0.342	0.469	0.159	0.136
Lingxia 35	0.485	0.064	0.938	0.909
Bamai 20	0.563	0.000	0.906	0.635
Longchun 35	0.485	1.000	0.834	0.460
1407	0.560	0.486	0.755	0.574
Ningchun 32	0.496	0.605	0.732	0.424
Yong 2563	0.403	0.472	0.642	0.416

Under drought stress, several traits showed high factor loadings in the composite indices, including aboveground phenotypic traits such as leaf area, plant height, and stomatal length, as well as root system architecture-related traits such as the cortical/stele area ratio, average root length, total root length, maximum root depth, and aerenchyma/cortical area ratio. Higher CI values indicate stronger drought tolerance, while lower CI values reflect weaker drought tolerance.

Membership function values for the composite indices were calculated for each cultivar under drought stress, as described in Equation 3. The weights of the composite indices were then determined using Equation 4, yielding values of 0.585, 0.173, 0.150, and 0.092 for the four indices, respectively (**Table 3**). Drought tolerance scores (D values) were calculated for each cultivar using Equation 5, and cultivars were ranked accordingly (**Table 5**).

**Table 5:** D values of each variety under drought stress.

	No. Variety	D values		No. Variety	D values
1	Ruichun1	0.927	15	Bamai 19	0.318
2	1538	0.361	16	Longchun 41	0.317
3	2038	0.476	17	L623	0.461
4	Ningchun 11	0.741	18	L622	0.452
5	9396	0.510	19	Yongliang 15	0.523
6	Longchun 30	0.454	20	Dingxi 49	0.182
7	Longchun 34	0.639	21	Dingxi 48	0.092
8	Linmai 33	0.351	22	Dingxi 40	0.304
9	Ningchun 16	0.656	23	Lingxia 35	0.490
10	Ningchun 4	0.578	24	Bamai 20	0.378
11	SM14	0.427	25	Longchun 35	0.519
12	Ningchun 57	0.666	26	1407	0.523
13	Ningchun 15	0.244	27	Ningchun 32	0.624
14	Ningchun 52	0.544	28	Yong 2563	0.552

**Figure 4:**
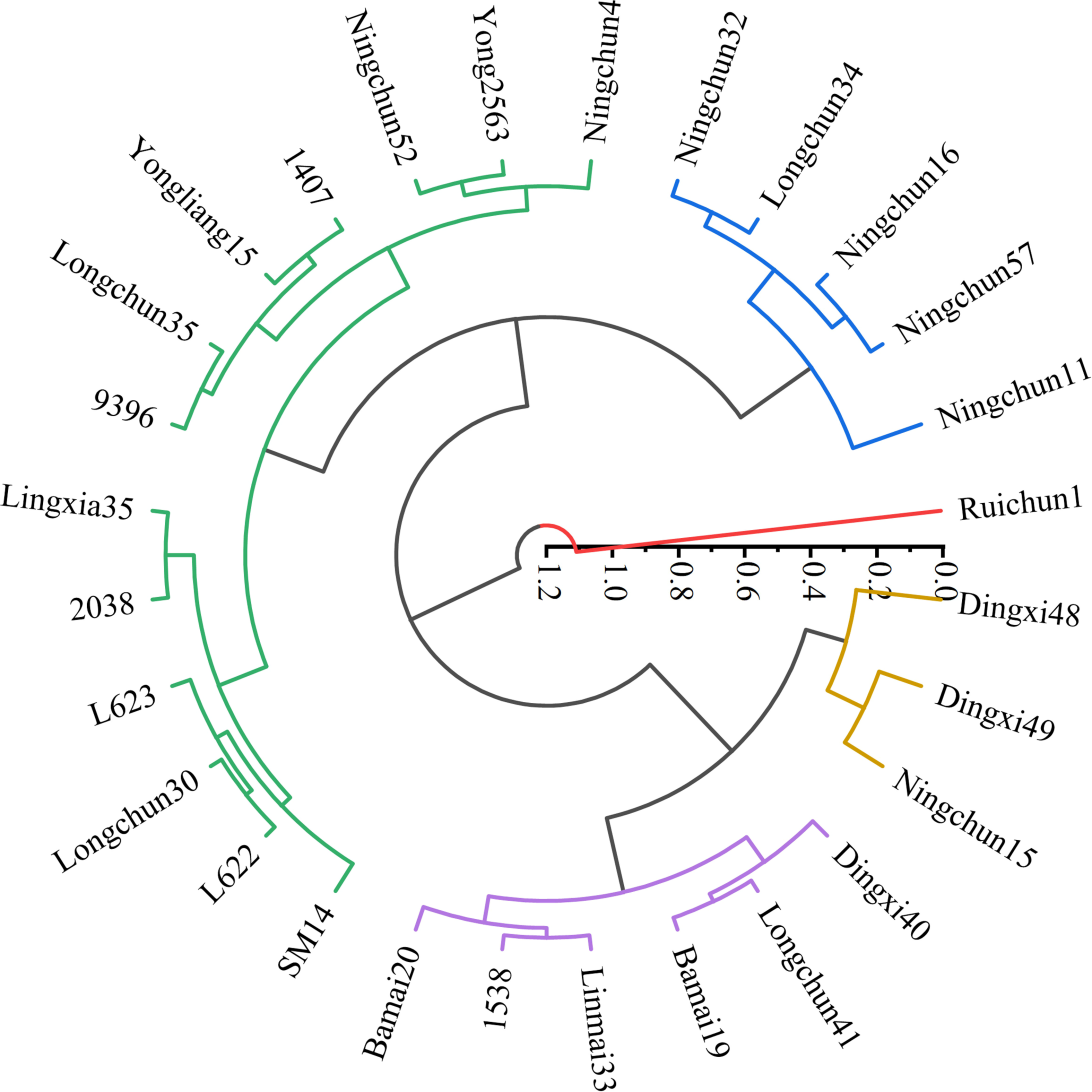
Hierarchical clustering analysis of D values for 28 spring wheat varieties under drought stress.

To further classify drought tolerance, Euclidean distance-based hierarchical clustering was applied to the D values. Under 10% PEG stress, with a Euclidean distance threshold of 0.840, the 28 cultivars were classified into five groups: drought-tolerant, relatively drought-tolerant, intermediate, relatively sensitive, and drought-sensitive. “Ruichun 1,” “Ningchun 11,” and “Ningchun 57” were classified as drought-tolerant cultivars, while “Dingxi 48” was identified as drought-sensitive (**Figure 4**). Compared to the sensitive cultivar “Dingxi 48,” the three drought-tolerant cultivars consistently exhibited higher drought tolerance coefficients for maximum root depth, convex hull area, plant height, leaf area, and stomatal density, as well as a lower cortical/stele area ratio. These findings indicate that both root and shoot traits contribute to their superior drought performance.

### 3.3 Potential phenotypic indicators for drought-tolerant genotypes

**Figure 5:**
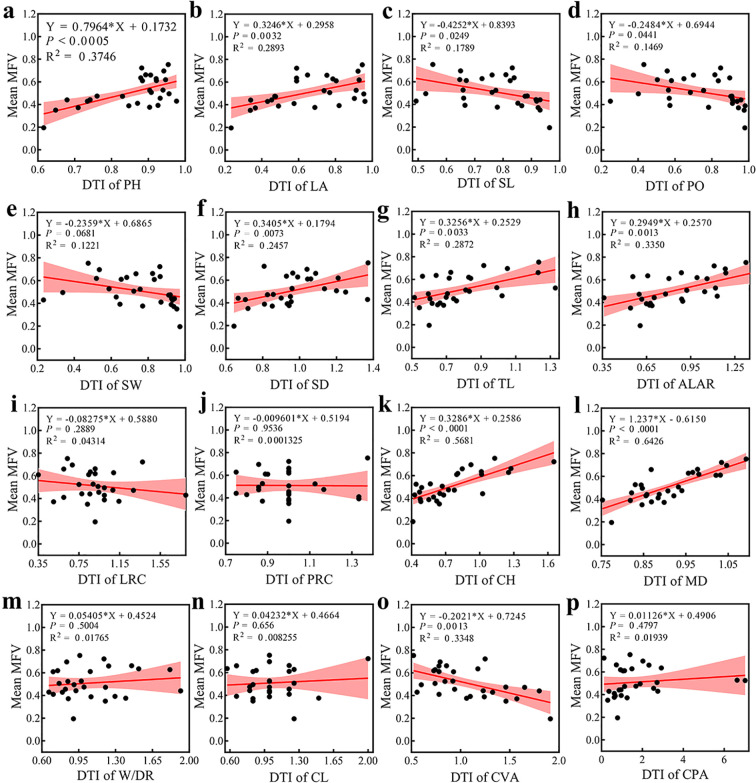
Linear model fitting under drought stress, illustrating the relationship between the average MFV and DTI for various seedling traits. MFV and DTI for various seedling traits. PH, Plant height; LA, Leaf area; ALAR, Total length of all roots; CH, Convex hull area; MD, Maximum root depth; CVA, Cortical/stele area.

Potential candidate phenotypic indicators for early-stage screening of drought-tolerant genotypes were identified by constructing simple linear regression models between the drought tolerance index (DTI) of each trait and the mean membership function value (MFV). As shown in **Figure 5**, under 10% PEG-induced osmotic stress, traits such as the cortical/stele area, stomatal length, stomatal width, and stomatal aperture exhibited negative correlations with increasing MFV. This negative association can be explained by adaptive responses: smaller stomatal traits reduce transpirational water loss, and a lower cortical/stele area decreases root metabolic costs, both of which contribute to improved whole-plant water use efficiency and drought tolerance. In contrast, plant height, leaf area, stomatal density, total root length, average root length, convex hull area, and maximum root depth showed positive correlations with increasing MFV. Specifically, the DTI of maximum root depth (*p* < 0.0001) exhibited the strongest correlation with mean MFV (R² = 0.629), followed by convex hull area (*p* < 0.0001), plant height (*p* < 0.001), average root length (*p* < 0.01), and cortical/stele area (*p* < 0.01), with corresponding coefficients of determination of 0.552, 0.351, 0.310, and 0.310. These results suggest that maximum root depth, convex hull area, and plant height serve as potential candidate traits for the seedling stage screening of drought tolerance in spring wheat at the seedling stage.

Moreover, the DTI of grain yield per plot showed significant correlations with the DTI of plant height, leaf area, stomatal length, stomatal width, stomatal density, convex hull area, maximum root depth, and cortical/stele area (*p* < 0.05) (**Supplementary Figure S2**). Among these, the DTI of maximum root depth (*p* < 0.0001) showed the strongest correlation with GYP (R² = 0.4392), followed by convex hull area (*p* < 0.001) and cortical/stele area (*p* < 0.01), with corresponding coefficients of determination of 0.3711 and 0.2365 (**Supplementary Figure S3**). These findings suggest that maximum root depth, convex hull area, and cortical/stele area are closely related to yield stability under drought stress, further highlighting their potential as candidate traits for drought-tolerant breeding in the seedling stage of spring wheat.

### 3.4 Correlations between aboveground traits and root system architecture

**Figure 6:**
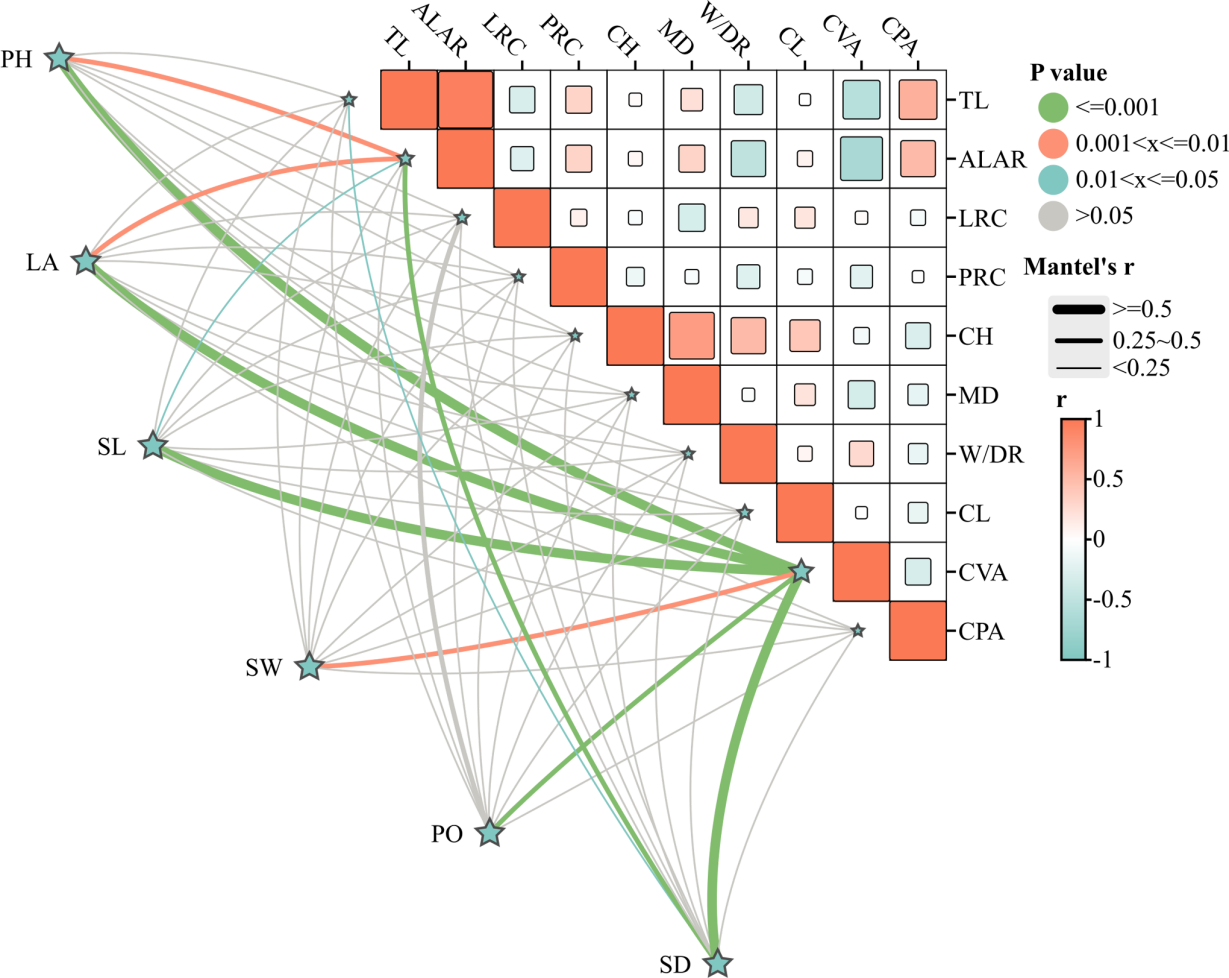
Correlation analysis of drought tolerance traits between aboveground and underground parts of spring wheat under 10% PEG stress. PH, Plant height; LA, Leaf area; SL, Stomatal length; SW, Stomatal width; PO, Stomatal aperture; SD, Stomatal density; TL, Total root length; ALAR, Total length of all roots; LRC, Number of lateral roots; PRC, Number of primary roots; CH, Convex hull area; MD, Maximum root depth; W/DR, Width/depth ratio; CL, Number of cortical layers; CVA, Cortical/stele area; CPA, Aerenchyma/cortical area.

Under 10% PEG induced drought stress, Spearman correlation analysis revealed significant associations between aboveground phenotypic traits and root architectural traits among different spring wheat cultivars (**Figure 6**). Specifically, plant height, leaf area, and stomatal density were significantly and positively correlated with root traits such as the average length of all roots and the cortical/stele area. In particular, the positive relationships between stomatal density and leaf area with cortical/stele area reflect a harmonized response between photosynthetic capacity and root anatomical structure. In addition, significant correlations were observed between root architectural traits and anatomical characteristics. Traits including total root length, average length of all roots, maximum root depth, and width/depth ratio were significantly associated with the aerenchyma/cortical area and cortical/stele area. Specifically, the aerenchyma/cortical area showed significant negative correlations with total root length, average length of all roots, and maximum root depth, whereas cortical/stele area exhibited significant positive correlations with these traits. Overall, the correlations between aboveground traits and root system architecture suggest that during drought adaptation, spring wheat enhances both root and shoot stress tolerance through a synchronized mechanism involving optimized deep root configuration and cortical tissue allocation.

### 3.5 Deep-root configuration traits of spring wheat under drought stress

**Figure 7:**
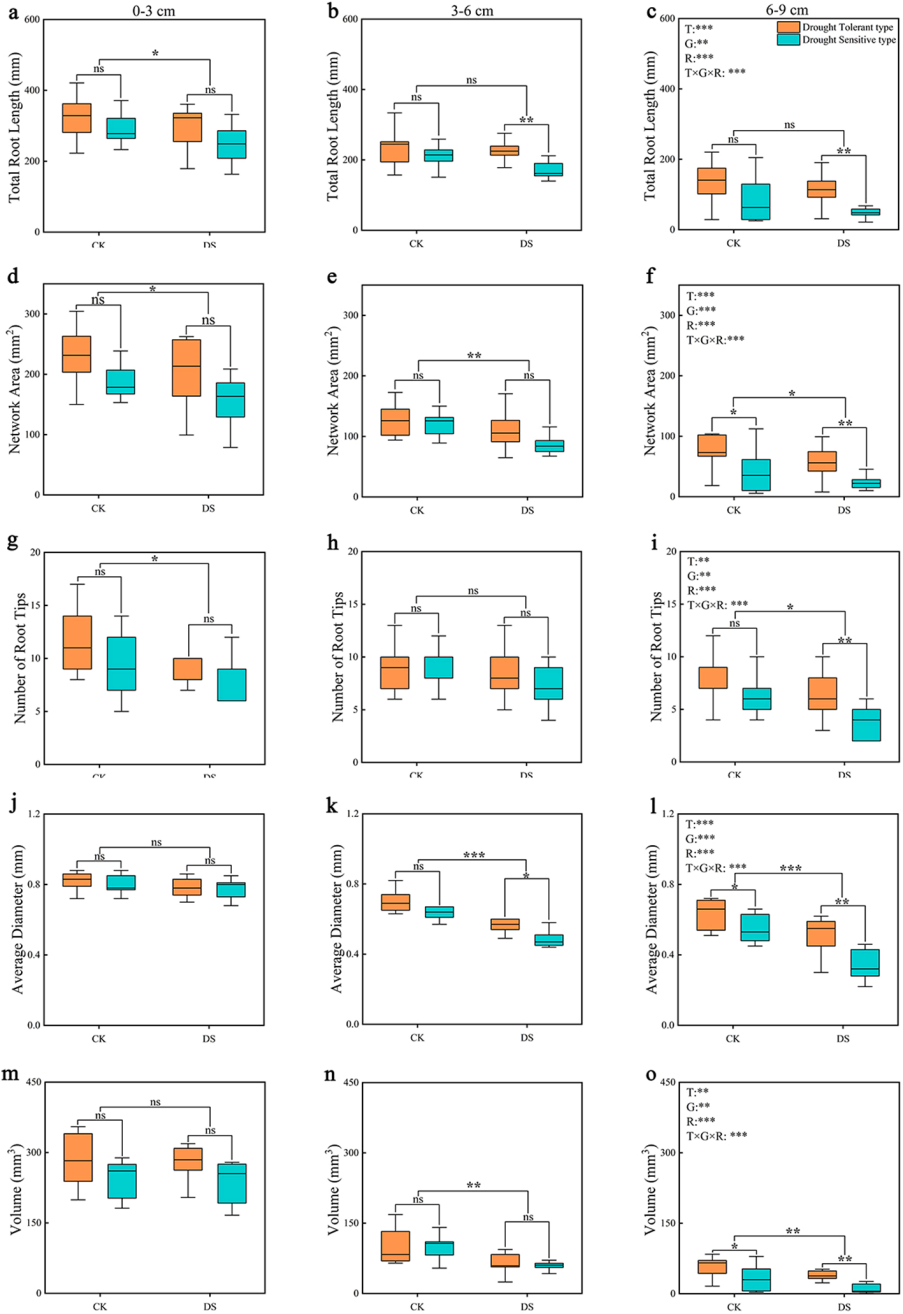
Phenotypic characteristics of root systems at different depths in tow spring wheat cultivars with significant responses under Drought stress. T: Treatment; G: Genotype; T×G: Genotype × Treatment interaction. *p< 0.05; **p< 0.01; ***p < 0.001; ns, Non-significant.

Based on the above results, two drought-tolerant cultivars (“Ruichun 1” and “Ningchun 57”) and two drought-sensitive cultivars (“Dingxi 48” and “Dingxi 49”) were selected for further investigation of root system stratification (**Figure 7**). In the 6–9 cm root zone, drought stress significantly reduced root volume and average root diameter (*p* < 0.01). However, drought-tolerant cultivars maintained larger root volume and diameter under stress, with mean values significantly higher than those of drought-sensitive cultivars by 62.158% and 29.688%, respectively (**Figures 7j–o**). In the 3–6 cm intermediate root zone, both traits were generally lower than in the 0–3 cm upper root zone, indicating that radial root growth and biomass allocation decrease with increasing root depth. Notably, in the intermediate root zone, the average root diameter of drought-tolerant cultivars remained significantly greater than that of sensitive cultivars (*p* < 0.001), with an increase of 21.023% (**Figures 7k–n**), This suggests that drought-tolerant genotypes are better able to maintain radial growth in intermediate-to-deep root segments. Furthermore, under drought stress in the 3–6 cm intermediate root zone, the drought-tolerant cultivar showed a lower cortical/stele area ratio, with a smaller increase compared to the sensitive cultivar. The increases were 12.740% and 36.633% for the drought-tolerant and sensitive cultivars, respectively (**Supplementary Figure S4a**). The smaller increase in the cortical/stele ratio in drought-tolerant cultivars is an adaptive response, reflecting reduced carbon allocation to cortical tissue. This likely lowers respiratory costs and reserves resources for root elongation. In contrast, sensitive cultivars exhibit a larger, less efficient increase.

In the 6–9 cm root zone, drought stress significantly reduced network area and root tip number compared with the control (*p* < 0.05). Nevertheless, under stress conditions, drought-tolerant cultivars maintained higher levels of total root length, network area, and root tip number, with mean values significantly exceeding those of drought-sensitive cultivars by 37.911%, 57.043%, and 44.262%, respectively (**Figures 7a–i**). In the 3–6 cm intermediate root zone, these three traits were generally lower than those in the 0–3 cm upper root zone. Even so, total root length of drought-tolerant cultivars in this root segment remained significantly higher than that of sensitive cultivars (*p<* 0.01), with an increase of 27.669% (**Figure 7b**). These results indicate that drought-tolerant genotypes possess a stronger capacity to sustain longitudinal root growth in intermediate and deep root layers.

A significant Treatment × Genotype × Root layer interaction was detected for all traits (*p<* 0.001), indicating that genotypic differences in osmotic stress response were dependent on root spatial distribution.

## 4 Discussion

### 4.1 Advantages of the paper-based high-throughput phenotyping platform

The paper-based high-throughput phenotyping platform effectively addresses key limitations of traditional methods, such as low efficiency, high destructiveness, and poor scalability in acquiring root drought-tolerance traits. As a result, it represents a core technical advantage of this study (Le Marie et al., 2014). This system enables the synchronized acquisition of root system architecture and aboveground traits during the germination stage for 28 spring wheat cultivars under controlled conditions. Each experiment processes hundreds of plants simultaneously, significantly increasing sample throughput and experimental efficiency for drought-tolerance screening.

In this system, roots grow along the plane of the filter paper, enabling high-resolution imaging and subsequent automated image analysis. The platform accurately extracts key root architectural parameters, including total root length, maximum rooting depth, convex hull area, and root tip number. It also supports combined analyses with aboveground traits, such as stomatal characteristics and leaf area. This root-shoot analysis allows for a comprehensive assessment of drought adaptation during the germination stage. Overall, the platform enables high-throughput phenotyping with fine spatial resolution and integrated root-shoot phenotypic characterization within a single experimental framework. This markedly enhances the capacity to evaluate and support data-driven assessments of drought tolerance traits at the early stage of wheat development.

However, compared to advanced three-dimensional imaging technologies like X-ray CT and MRI, the paper-based platform has limitations in spatial resolution and environmental realism. Despite these limitations, it offers a clear advantage in cost-effectiveness for early-stage breeding applications. While 3D imaging can reconstruct complete root systems in soil or soil-like media, it requires expensive equipment, has operational complexity, and offers limited throughput, making it impractical for routine screening of dozens to hundreds of genotypes (Metzner et al., 2015). In contrast, the paper-based system is inexpensive, easy to construct and operate, and its imaging and analysis workflows are easily standardized. This makes it ideal for parallel comparisons of large germplasm collections under uniform PEG-induced drought stress (Le Marie et al., 2014). It is important to recognize the inherent limitations of using PEG-6000 to simulate drought stress in this artificial system. Osmotic potential, induced by PEG, affects water availability through solute concentration, while matric potential, which prevails in soils, arises from capillary and adsorptive forces within the soil matrix. These forces influence water mobility and root-soil contact. While PEG effectively imposes uniform osmotic stress, it cannot replicate the full physiological and physical complexity of soil-based drought due to the absence of matric potential effects (Tuberosa, 2012; Richard et al., 2015). Therefore, the results should be interpreted as indicators of genotypic “potential” rather than absolute field values. These findings reflect genotypic potential, which must be validated in field conditions before extrapolation; direct translation to field conditions is not advisable without further testing. Despite these limitations, evidence supports the translatability of these results to field conditions. Genotypic rankings for root architectural traits and early vigor often show significant correlations between controlled-environment platforms and soil-grown plants (Trachsel et al., 2011). Specifically, root depth potential at the seedling stage and the associated shoot-root adjustments identified in high-throughput systems reliably predict drought avoidance strategies and biomass accumulation in adult plants under field conditions (Tuberosa, 2012; Kathirvelu et al., 2025). Thus, while our findings require field-scale validation to account for dynamic root-soil interactions, the paper-based platform remains a robust and biologically relevant tool for the preliminary screening of drought-resilient germplasm in spring wheat.

Finally, by combining the paper-based high-throughput platform with a multi-trait comprehensive evaluation framework, this study advances from “tool to system,” building on previous research. Earlier studies have utilized paper-based or growth-bag systems, such as germination pouches and Rhizoslides, to measure seedling root structural traits (e.g., root length, root number, and root angle) in a high-throughput manner. These studies also explored the relationships between early root architecture and stress tolerance (Atkinson et al., 2015). However, these platforms typically focus on a limited set of structural traits and rarely integrate root anatomical characteristics, aboveground traits, and overall drought indices into a unified evaluation framework. In this study, PEG-simulated drought stress was introduced into the paper-based phenotyping system, along with concurrent measurements of root anatomical traits and aboveground indicators such as stomatal density and leaf area. Specifically, root anatomical traits were measured in different root segments: 0–3 cm, 3–6 cm, and 6–9 cm from the root tip. This approach enabled the incorporation of longitudinal anatomical variation along the primary seminal root into the evaluation system. Through principal component analysis, the membership function method, and D-value computation, a multi-trait comprehensive evaluation system was established. Although these analytical procedures are established methods widely used in crop drought-tolerance evaluation, the novelty of this study lies in the type and combination of traits incorporated into this framework rather than in the statistical methods themselves. Principal component analysis, the membership function method, and D-value computation were then used to establish a multi-trait comprehensive evaluation system. Although the analytical methods used in this study are well-established in crop drought-tolerance evaluation, the novelty lies in the integration of traits within this framework, rather than the statistical methods themselves. This system combines root architecture, anatomical characteristics, aboveground growth, and drought tolerance indices. To our knowledge, this is the first study in spring wheat to explicitly integrate stratified root anatomical traits, whole-root architecture, and shoot traits into a PCA-based D-value screening framework for seedling-stage drought tolerance. This integration allows the framework to capture coupled deep-root configuration and cortical allocation strategies, which are often overlooked in conventional screening studies that focus primarily on aboveground traits, germination indices, or simple root length measurements. By extending a single-phenotype platform into a multi-trait unified evaluation framework, the paper-based approach retains its high-throughput operational advantages while demonstrating strong methodological integration and innovation. This provides a more practical solution for drought tolerance research in spring wheat. Within this integrated framework, we employed the D-value method to combine multiple trait categories into a single index. The D-value method was selected because it provides contribution-based weighting, reduces dimensionality, and generates a single ranking index. Its advantages over the geometric mean or TOPSIS include objectivity and computational simplicity. However, limitations include linearity assumptions, sensitivity to sample size, and population-specific results. Therefore, it is suitable for relative screening within a given genotype set but not for absolute comparisons across independent experiments. As a validation, we compared the D-value rankings with those from equal-weighted membership function and geometric mean using the same dataset. The Spearman rank correlation coefficients were 0.63 (*p* < 0.001) and 0.57 (*p* < 0.001), confirming moderate to strong consistency (**Supplementary Table S3**).

### 4.2 Coordinated root-shoot responses under drought stress in spring wheat

This study systematically examines the coupled response patterns between aboveground traits and root characteristics of spring wheat under drought stress. Correlation analyses revealed significant positive relationships between aboveground phenotypic traits (e.g., plant height, leaf area, and stomatal density) and root architectural traits (e.g., total root length and maximum root depth). This coupling highlights the complementary functioning of both systems. Aboveground, stomatal structural traits-such as stomatal length, stomatal width, stomatal aperture, and stomatal density-regulate gas exchange and stomatal conductance, balancing transpirational water loss with carbon assimilation (Rahnama et al., 2010). At the same time, belowground adaptation enhances spatial exploration and metabolic efficiency. Traits such as greater rooting depth potential and a lower cortical/stele area (CVA) maximize resource acquisition from deeper soil zones while minimizing respiratory maintenance costs (Vanhees et al., 2020). Together, these “demand-side” aboveground regulatory mechanisms and belowground capacities ensure the plant optimizes water-use efficiency while maintaining the growth vigor necessary to fuel physiological defenses under osmotic stress. Additionally, the drought tolerance index (DTI) for these root architectural traits correlates significantly with yield stability under drought conditions. Specifically, maximum root depth and convex hull area were highly correlated with the DTI of grain yield per plot, suggesting that these traits enhance yield stability in drought-prone environments.

Crucially, the synergy between these traits forms a reciprocal support mechanism that ensures whole-plant survival. In this feedback loop, the root system adjusts its structural and anatomical traits to enhance water and nutrient acquisition, providing the necessary hydraulic support to maintain leaf turgor and photosynthetic activity in the shoot (Macinnis-Ng, 2024). In turn, adaptive changes in aboveground traits-particularly the regulation of stomatal density and aperture-minimize excessive water loss and maintain a stable internal water status. This stability, coupled with a continuous supply of assimilates from the leaves, provides the energy and hydraulic conditions necessary for continued root elongation and metabolic function (Ramachandra et al., 2024). This mutualistic relationship allows the plant to sustain a dynamic equilibrium between belowground resource supply and aboveground metabolic demand within a constrained environment.

Ultimately, the results of this study suggest that the coupling between roots and shoots in spring wheat aligns with a unified strategy for drought adaptation. It is not merely a collection of independent responses, but reflects a holistic evolutionary balance in which the plant operates as a cohesive adaptive whole to maintain physiological stability. Instead of viewing the responses of individual organs in isolation, our findings demonstrate that drought tolerance in spring wheat seedlings is facilitated by the coordinated synergy between aboveground and belowground components. By examining this coupling, we highlight the importance of the functional equilibrium between evaporative demand from the atmosphere and water acquisition by the roots as a key component of drought resilience. This reinforces the value of adopting a whole-plant perspective to better understand the unified adaptive mechanisms underlying drought tolerance during the early seedling stages.

### 4.3 Deep root configuration and cortical allocation enhance drought adaptation

This study emphasizes the critical role of deep root configuration in drought adaptation of spring wheat. Stratified root analyses revealed that in the intermediate and deep zones of the growth medium (6–9 cm), drought-tolerant cultivars significantly outperformed drought-sensitive cultivars in key traits such as total root length, root tip number, network area, and convex hull area. This result aligns with previous studies suggesting that drought-tolerant cultivars extend their root systems deeper into the growth medium to optimize spatial occupancy under osmotic stress (Narayanan et al., 2014). Furthermore, tolerant wheat seedlings maintain higher root length density and more root tips in the lower segments of the stratified root system (Beyer et al., 2019), which is consistent with the observed growth advantage in the deeper zones of the pouch in this study. Interestingly, while drought-tolerant genotypes maintained or even increased total root length under stress, they exhibited a significant reduction in average root diameter. This suggests a trade-off between root thickness and longitudinal expansion. By producing thinner roots, tolerant cultivars may reduce the construction cost per unit length, facilitating greater root elongation under osmotic stress (Lynch et al., 2021). This strategy likely enables the plant to allocate limited resources toward downward root elongation and spatial exploration of deeper zones, rather than lateral thickening. As previous studies have shown, this compensatory relationship between root diameter and length is essential for maximizing exploration under resource-limited conditions (Lynch, 2013). Additionally, this adjustment may reduce the respiratory burden of root exploration (Lynch et al., 2021). Therefore, the deep root advantage observed in this study is not merely due to increased biomass but reflects an anatomical optimization prioritizing root depth through the strategic reduction of root diameter.

In this experimental setup, water potential remains uniform throughout the pouch. As a result, the observed deep root advantage primarily reflects intrinsic growth vigor and expansion potential, rather than a direct function of accessing deeper water resources. In this controlled context (**Figure 7**), a highly significant Treatment × Genotype × Root layer interaction (*p* < 0.001) was observed for five traits. This indicates that genotypic responses to osmotic stress vary spatially across root zones. Because treatment effects depend on both genotype and root position, the observed differences cannot be solely attributed to constitutive root vigor under control conditions. Instead, the significant three-way interaction suggests genotype-specific plasticity in root system reconfiguration under stress. Specifically, drought-tolerant cultivars maintained longitudinal growth and favorable cortical allocation in deeper layers, whereas sensitive genotypes showed greater reductions. These results suggest that osmotic stress tolerance is expressed through spatially coupled root plasticity, rather than merely through baseline growth differences. Therefore, these anatomical adjustments should primarily be interpreted as adaptive plastic responses to osmotic stress, superimposed on fixed constitutive structural differences among cultivars.

Increased aerenchyma formation may represent an adaptive response to drought stress, as it has been reported to reduce living cortical tissue and lower root respiratory costs (Zhu et al., 2010; Jaramillo et al., 2013). This adjustment likely enables roots to maintain essential functions under water deficit with reduced carbon investment. The negative correlations between aerenchyma/cortical area and root length and depth suggest a trade-off between anatomical modification and root elongation in this system. Thus, an increased aerenchyma proportion may contribute to drought adaptation by improving root metabolic efficiency, rather than necessarily promoting greater root elongation or depth (Chimungu et al., 2015).

Drought-tolerant cultivars maintained larger root volume and drought-associated cortical patterns. These traits likely enhance metabolic efficiency and the ability of roots to explore the growth medium. Previous research has shown that efficient cortical arrangements reduce the respiratory costs of environmental exploration (Zhu et al., 2010; Chimungu et al., 2015), facilitating deeper penetration under stress. In contrast, sensitive genotypes showed weaker maintenance of these cortical patterns (Lynch, 2013). Furthermore, maintaining specific root volumes in distant zones appears essential for sustaining growth vigor under limited water availability, while less resilient varieties exhibited weaker structural adjustments.

To translate this structural advantage into actionable breeding strategies, this study integrates the synergistic mechanisms of deep root configuration and cortical allocation into a comprehensive evaluation framework. D-value and cluster analyses confirmed the importance of these indicators in distinguishing drought tolerance types. This approach effectively links structure, anatomy, and combined drought tolerance, offering significant methodological advantages and practical application value. Among the evaluated traits, maximum root depth, convex hull area, and several cortex-related anatomical indicators showed strong explanatory power for the comprehensive drought tolerance index (DTI) in multivariate regression models. These traits are physiologically relevant and exhibit stable discriminatory capacity from a statistical perspective. More importantly, they can be measured rapidly and reproducibly using the paper-based high-throughput phenotyping platform, facilitating standardized screening of large germplasm collections at early seedling stages. This study translates the interaction between deep root deployment and cortical optimization into a set of quantifiable and high-throughput measurable candidate indicators for drought tolerance screening in spring wheat at the seedling stage under controlled environmental conditions. It establishes a potential framework for the preliminary screening of drought tolerance at early growth stages. These traits are promising indicators that can aid in selecting adaptive genotypes during the early phases of breeding programs. However, it is important to note that these findings were obtained under PEG-induced osmotic stress, rather than under soil-based field drought conditions. Therefore, further validation is required to assess their reliability and contribution to final yield stability across various field environments and growing seasons before they can be used in practical breeding programs. Additionally, the relatively small panel size (28 cultivars) may limit the generalizability of these findings. Although the selected cultivars represent typical spring wheat germplasm from the arid and semi-arid regions of northwestern China, future studies should include larger, more diverse populations to confirm the trait associations observed here.

## 5 Conclusion

This study utilized a paper-based high-throughput phenotyping platform to measure root system architecture, root anatomical traits, and aboveground characteristics in spring wheat seedlings under PEG-induced osmotic stress. The platform provided a controlled environment for early-stage genotypic potential assessment. However, it is important to note that this system does not directly replicate field drought conditions. Despite this limitation, a relationship between field yield and the drought tolerance index (DTI) of both root and aboveground traits was observed, emphasizing yield stability under drought stress. Future studies should further validate this relationship under real field conditions, which will be crucial for translating these findings into practical breeding applications. The key findings are as follows: First, combining the D-value with cluster analysis classified the 28 cultivars into five drought tolerance groups. “Ruichun 1,” “Ningchun 11,” and “Ningchun 57” were identified as drought-tolerant, while “Dingxi 48” was classified as drought-sensitive. Second, maximum root depth, convex hull area, and plant height demonstrated substantial explanatory power for the D-value (R² = 0.629, 0.552, and 0.351, respectively), identifying them as core candidate traits for seedling-stage screening. These traits were also closely correlated with yield stability under drought stress, suggesting their potential as candidate traits for drought tolerance breeding in spring wheat. Third, drought-tolerant cultivars exhibited greater total root length, root tip number, and convex hull area in the 6–9 cm deep root zone, forming a coupled structural pattern characterized by deep root configuration and optimized cortical allocation. Fourth, significant positive correlations were observed between aboveground traits (plant height, leaf area, stomatal density) and root traits (total root length, average root length, maximum root depth), indicating that spring wheat seedlings maintain a functional balance between root and shoot growth under drought stress. Overall, deep and extensive root deployment, combined with low-cost, high-efficiency cortical organization, constitutes a key trait combination linked to drought adaptation in spring wheat seedlings. These findings provide a theoretical foundation for incorporating deep rooting capacity and cortical optimization into high-throughput screening procedures. However, these traits must undergo rigorous field validation before being considered for breeding drought-resilient cultivars.

## Data Availability

The datasets used during the current study are available from the corresponding author on reasonable request.

## Supplementary material

Supplementary Table S1 List of the 28 spring wheat varieties used in this study.

Supplementary Table S2 Monthly rainfall and irrigation amounts during the 2025 spring wheat growing season.

Supplementary Table S3 Cultivar rankings obtained by D-value, EW-MFV, and geometric mean. Spearman’s rank correlation coefficients: ρ = 0.63 (D vs. EW-MFV, *p* < 0.001) and ρ = 0.57 (D vs. GM, *p* < 0.001).

Supplementary Table S4 Grain yield-based drought tolerance coefficients of 28 spring wheat cultivars under drought stress in 2025.

Supplementary Figure S1 Flowchart showing the calculation of the D value from root and shoot traits. Steps: (1) Trait measurement; (2) Drought tolerance coefficients; (3) PCA; (4) Membership function and weighting; (5) D value; (6) Drought tolerance classification.

Supplementary Figure S2 Correlation analysis between plot grain yield and seedling stage drought stress traits. PH, Plant height; LA, Leaf area; SL, Stomatal length; SW, Stomatal width; PO, Stomatal aperture; SD, Stomatal density; TL, Total root length; ALAR, Total length of all roots; CH, Convex hull area; MD, Maximum root depth; W/DR, Width/depth ratio; CL, Number of cortical layers; CVA, Cortical/stele area; CPA, Aerenchyma/cortical area; GYP, Grain yield per plot.

Supplementary Figure S3 Linear model fitting under drought stress, illustrating the relationship between the grain yield per plot drought tolerance index and the drought tolerance index of various seedling traits. PH, Plant height; LA, Leaf area; SL, Stomatal length; SW, Stomatal width; SD, Stomatal density; CH, Convex hull area; MD, Maximum root depth; CVA, Cortical/stele area; GYP, Grain yield per plot.

Supplementary Figure S4 Root anatomical characteristics of two spring wheat cultivars with significant responses in the 6–9 cm depth zone of growth pouches under drought stress. * *p<* 0.05; ** *p<* 0.01; *** *p* < 0.001; ns, Non-significant.

## References

[B1] Adeleke E. Millas R. McNeal W. Faris J. Taheri A. (2020). Variation analysis of root system development in wheat seedlings using root phenotyping system. Agronomy 10, 206. doi: 10.3390/agronomy10020206 30654563

[B2] Atkinson J. A. Pound M. P. Bennett M. J. Wells D. M. (2019). Uncovering the hidden half of plants using new advances in root phenotyping. Curr. Opin. Biotechnol. 55, 1–8. doi: 10.1016/j.copbio.2018.06.002 30031961 PMC6378649

[B3] Atkinson J. A. Wingen L. U. Griffiths M. Pound M. P. Gaju O. Foulkes M. J. . (2015). Phenotyping pipeline reveals major seedling root growth QTL in hexaploid wheat. J. Exp. Bot. 66, 2283–2292. doi: 10.1093/jxb/erv006 25740921 PMC4407652

[B4] Beyer S. Daba S. Tyagi P. Bockelman H. Brown-Guedira G. IWGSC . (2019). Loci and candidate genes controlling root traits in wheat seedlings: a wheat root GWAS. Funct. Integr. Genomics 19, 91–107. doi: 10.1007/s10142-018-0630-z 30151724

[B5] Chimungu J. G. Maliro M. F. A. Nalivata P. C. Kanyama-Phiri G. Brown K. M. Lynch J. P. (2015). Utility of root cortical aerenchyma under water limited conditions in tropical maize (Zea mays L.). Field Crops Res. 171, 86–98. doi: 10.1016/j.fcr.2014.10.009

[B6] Ding L. Li Y. Wang Y. Gao L. Wang M. Chaumont F. . (2016). Root ABA accumulation enhances rice seedling drought tolerance under ammonium supply: interaction with aquaporins. Front. Plant Sci. 7, 1–10. doi: 10.3389/fpls.2016.01206 27559341 PMC4979525

[B7] Gifford M. L. Xu G. Dupuy L. X. Vissenberg K. Rebetzke G. (2024). Root architecture and rhizosphere-microbe interactions. J. Exp. Bot. 75, 503–507. doi: 10.1093/jxb/erad488 38197460 PMC10773993

[B8] Gill T. Gill S. K. Saini D. K. Chopra Y. De Koff J. P. Sandhu K. S. (2022). A comprehensive review of high throughput phenotyping and machine learning for plant stress phenotyping. Phenomics 2, 156–183. doi: 10.1007/s43657-022-00048-z 36939773 PMC9590503

[B9] Guo C. Zhang K. Sun H. Zhu L. Zhang Y. Wang G. . (2025). Root cortical senescence enhances drought tolerance in cotton. Plant Cell Environ. 48, 615–633. doi: 10.1111/pce.15161 39300935

[B10] Henry C. M. Deacon J. W. (1981). Natural (non-pathogenic) death of the cortex of wheat and barley seminal roots, as evidenced by nuclear staining with acridine orange. Plant Soil 60, 255–274. doi: 10.1007/BF02374110 30311153

[B11] Holz M. Zarebanadkouki M. Benard P. Hoffmann M. Dubbert M. (2024). Root and rhizosphere traits for enhanced water and nutrients uptake efficiency in dynamic environments. Front. Plant Sci. 15, 1383373. doi: 10.3389/fpls.2024.1383373 39145194 PMC11322101

[B12] Jaramillo R. E. Nord E. A. Chimungu J. G. Brown K. M. Lynch J. P. (2013). Root cortical burden influences drought tolerance in maize. Ann. Bot. 112, 429–437. doi: 10.1093/aob/mct069 23618897 PMC3698389

[B13] Kathirvelu D. Singh B. Ramasamy R. Krishna H. Vishwakarma C. Pandey R. . (2025). Association of seedling root traits with field level drought tolerance in wheat (Triticum aestivum). Plant Physiol. Rep. 30, 569–581. doi: 10.1007/s40502-025-00884-x 30311153

[B14] Le Marie C. Kirchgessner N. Marschall D. Walter A. Hund A. (2014). Rhizoslides: paper-based growth system for non-destructive, high throughput phenotyping of root development by means of image analysis. Plant Methods 10, 13. doi: 10.1186/1746-4811-10-13 25093035 PMC4105838

[B15] Li C. Li L. Reynolds M. P. Wang J. Chang X. Mao X. . (2021). Recognizing the hidden half in wheat: root system attributes associated with drought tolerance. J. Exp. Bot. 72, 5117–5133. doi: 10.1093/jxb/erab124 33783492

[B16] Lynch J. P. (2013). Steep, cheap and deep: an ideotype to optimize water and N acquisition by maize root systems. Ann. Bot. 112, 347–357. doi: 10.1093/aob/mcs293 23328767 PMC3698384

[B17] Lynch J. P. Strock C. F. Schneider H. M. Sidhu J. S. Ajmera I. Galindo-Castañeda T. . (2021). Root anatomy and soil resource capture. Plant Soil 466, 21–63. doi: 10.1007/s11104-021-05010-y 30311153

[B18] Ma J. Niklas K. J. Liu L. Fang Z. Li Y. Shi P. (2022). Tree size influences leaf shape but does not affect the proportional relationship between leaf area and the product of length and width. Front. Plant Sci. 13, 850203. doi: 10.3389/fpls.2022.850203 35755713 PMC9221507

[B19] Macinnis-Ng C. (2024). Transport of water to leaves implies whole-plant coordination of hydraulic and photosynthetic traits. New Phytol. 244, 1681–1683. doi: 10.1111/nph.20045 39135393

[B20] Metzner R. Eggert A. Van Dusschoten D. Pflugfelder D. Gerth S. Schurr U. . (2015). Direct comparison of MRI and X-ray CT technologies for 3D imaging of root systems in soil: potential and challenges for root trait quantification. Plant Methods 11, 17. doi: 10.1186/s13007-015-0060-z 25774207 PMC4359488

[B21] Nagel K. A. Putz A. Gilmer F. Heinz K. Fischbach A. Pfeifer J. . (2012). GROWSCREEN-rhizo is a novel phenotyping robot enabling simultaneous measurements of root and shoot growth for plants grown in soil-filled rhizotrons. Funct. Plant Biol. 39, 891–904. doi: 10.1071/FP12023 32480839

[B22] Narayanan S. Mohan A. Gill K. S. Prasad P. V. V. (2014). Variability of root traits in spring wheat germplasm. PloS One 9, e100317. doi: 10.1371/journal.pone.0100317 24945438 PMC4063797

[B23] Ouyang W. Yin X. Yang J. Struik P. C. (2020). Comparisons with wheat reveal root anatomical and histochemical constraints of rice under water-deficit stress. Plant Soil 452, 547–568. doi: 10.1007/s11104-020-04581-6 30311153

[B24] Pathoumthong P. Zhang Z. Roy S. J. El Habti A. (2023). Rapid non-destructive method to phenotype stomatal traits. Plant Methods 19, 36. doi: 10.1186/s13007-023-01016-y 37004073 PMC10064510

[B25] Rahnama A. Fakhri S. Meskarbashee M. (2019). Root growth and architecture responses of bread wheat cultivars to salinity stress. Agron. J. 111, 2991–2998. doi: 10.2134/agronj2018.12.0795

[B26] Rahnama A. Hosseinalipour B. Farrokhian Firouzi A. Tom Harrison M. Ghorbanpour M. (2024). Root architecture traits and genotypic responses of wheat at seedling stage to water-deficit stress. Cereal Res. Commun. 52, 1499–1510. doi: 10.1007/s42976-023-00481-4 30311153

[B27] Rahnama A. James R. A. Poustini K. Munns R. (2010). Stomatal conductance as a screen for osmotic stress tolerance in durum wheat growing in saline soil. Funct. Plant Biol. 37, 255–263. doi: 10.1071/FP09148 38477348

[B28] Rahnama A. Munns R. Poustini K. Watt M. (2011). A screening method to identify genetic variation in root growth response to a salinity gradient. J. Exp. Bot. 62, 69–77. doi: 10.1093/jxb/erq359 21118825

[B29] Ramachandra A. Vijayaraghavareddy P. Purushothama C. Nagaraju S. Sreeman S. (2024). Decoding stomatal characteristics regulating water use efficiency at leaf and plant scales in rice genotypes. Planta 260, 56–71. doi: 10.1007/s00425-024-04488-x 39039321

[B30] Richard C. Hickey L. T. Fletcher S. Jennings R. Chenu K. Christopher J. T. (2015). High-throughput phenotyping of seminal root traits in wheat. Plant Methods 11, 13–24. doi: 10.1186/s13007-015-0055-9 25750658 PMC4351910

[B31] Trachsel S. Kaeppler S. M. Brown K. M. Lynch J. P. (2011). Shovelomics: high throughput phenotyping of maize (Zea mays L.) root architecture in the field. Plant Soil 341, 75–87. doi: 10.1007/s11104-010-0623-8 30311153

[B32] Tuberosa R. (2012). Phenotyping for drought tolerance of crops in the genomics era. Front. Physiol. 3, 347. doi: 10.3389/fphys.2012.00347 23049510 PMC3446691

[B33] Uga Y. Sugimoto K. Ogawa S. Rane J. Ishitani M. Hara N. . (2013). Control of root system architecture by DEEPER ROOTING 1 increases rice yield under drought conditions. Nat. Genet. 45, 1097–1102. doi: 10.1038/ng.2725 23913002

[B34] Vadez V. Grondin A. Chenu K. Henry A. Laplaze L. Millet E. J. . (2024). Crop traits and production under drought. Nat. Rev. Earth Environ. 5, 211–225. doi: 10.1038/s43017-023-00514-w 37880705

[B35] Vanhees D. J. Loades K. W. Bengough A. G. Mooney S. J. Lynch J. P. (2020). Root anatomical traits contribute to deeper rooting of maize under compacted field conditions. J. Exp. Bot. 71, 4243–4257. doi: 10.1093/jxb/eraa165 32420593 PMC7337194

[B36] Wang L. Chang C. (2024). Stomatal improvement for crop stress resistance. J. Exp. Bot. 75, 1823–1833. doi: 10.1093/jxb/erad477 38006251

[B37] Yamauchi T. Pedersen O. Nakazono M. Tsutsumi N. (2021). Key root traits of Poaceae for adaptation to soil water gradients. New Phytol. 229, 3133–3140. doi: 10.1270/jsbbs.20119 33222170 PMC7986152

[B38] Zhu J. Brown K. M. Lynch J. P. (2010). Root cortical aerenchyma improves the drought tolerance of maize (Zea mays L.). Plant Cell Environ. 33, 740–749. doi: 10.1111/j.1365-3040.2009.02099.x 20519019

