# Supplementary material for: Drought adaptation in spring wheat seedlings relies on coordinated deep root architecture and cortical tissue allocation
Source: Front Plant Sci. 2026 Jun 8;17:1846481. doi: 10.3389/fpls.2026.1846481 (PMC13285027; doi:10.3389/fpls.2026.1846481)
Supplement: Supplementary Table S1 — List of the 28 spring wheat varieties used in this study. [file Table1.docx]

**Table S1** List of the 28 spring wheat varieties used in this study.

| Variety | registration number | Variety | registration number |
| --- | --- | --- | --- |
| Ruichun1 | Gan Shen Mai 20230008 | Bamai 19 | Meng Shen Mai 2023008 |
| 1538 |  | Longchun 41 | Gan Shen Mai 20200002 |
| 2038 |  | L623 | Gan Shen Mai 20241002 |
| Ningchun 11 | DB64/T 082-1993 | L622 | Gan Shen Mai 20230009 |
| 9396 |  | Yongliang 15 | DB64/T 330-2004 |
| Longchun 30 | Gan Shen Mai 2013004 | Dingxi 49 | Gan Shen Mai 20210001 |
| Longchun 34 | Gan Shen Mai 2015002 | Dingxi 48 | DB62/T 4373‑2021 |
| Linmai 33 | Gan Shen Mai 2007001 | Dingxi 40 | Guo Shen Mai  2009032 |
| Ningchun 16 | Ning Shen Mai 9201 | Lingxia 35 | Guo Shen Mai 2013003 |
| Ningchun 4 | Ning Zhong Shen 8101 | Bamai 20 | Meng Shen Mai 2023007 |
| SM14 |  | Longchun 35 | Guo Shen Mai 2017005 |
| Ningchun 57 | Ning Shen Mai 8101 | 1407 |  |
| Ningchun 15 | Ning Shen Mai 20250001 | Ningchun 32 | Ning Shen Mai 200201 |
| Ningchun 52 | Ning Shen Mai 2012001 | Yong 2563 |  |
