# Supplementary material for: Drought adaptation in spring wheat seedlings relies on coordinated deep root architecture and cortical tissue allocation
Source: Front Plant Sci. 2026 Jun 8;17:1846481. doi: 10.3389/fpls.2026.1846481 (PMC13285027; doi:10.3389/fpls.2026.1846481)
Supplement: Supplementary Table S2 — Monthly rainfall and irrigation amounts during the 2025 spring wheat growing season. [file Table2.docx]

**Table S2** Monthly Rainfall and Irrigation Amounts during the 2025 spring wheat growing season.

| Months | Rainfall (mm) | Non-stress treatment Irrigation (mm) | Drought stress treatment Irrigation (mm) |
| --- | --- | --- | --- |
| April | 3.2 | 35 | 20 |
| May | 23.4 | 160 | 80 |
| June | 61.0 | 120 | 60 |
| July | 96.4 | 0 | 0 |
