# Supplementary material for: Drought adaptation in spring wheat seedlings relies on coordinated deep root architecture and cortical tissue allocation
Source: Front Plant Sci. 2026 Jun 8;17:1846481. doi: 10.3389/fpls.2026.1846481 (PMC13285027; doi:10.3389/fpls.2026.1846481)
Supplement: Supplementary Table S3 — Cultivar rankings obtained by D-value, EW-MFV, and geometric mean. Spearman’s rank correlation coefficients: ρ = 0.63 (D vs. EW-MFV, p < 0.001) and ρ = 0.57 (D vs. GM, p < 0.001). [file Table3.docx]

**Table S3** Cultivar rankings obtained by D-value, EW-MFV, and geometric mean.

| Cultivar | D values | Rank | EW-MFV | Rank | GM | Rank |
| --- | --- | --- | --- | --- | --- | --- |
| Ruichun1 | 0.927 | 1 | 0.893 | 1 | 0.878 | 1 |
| Ningchun 11 | 0.741 | 2 | 0.528 | 13 | 0.462 | 15 |
| Ningchun 57 | 0.666 | 3 | 0.713 | 3 | 0.696 | 3 |
| Ningchun 16 | 0.656 | 4 | 0.495 | 16 | 0.263 | 22 |
| Longchun 34 | 0.639 | 5 | 0.739 | 2 | 0.721 | 2 |
| Ningchun 32 | 0.624 | 6 | 0.564 | 11 | 0.556 | 10 |
| Ningchun 4 | 0.578 | 7 | 0.452 | 18 | 0.394 | 17 |
| Yong 2563 | 0.552 | 8 | 0.483 | 17 | 0.477 | 14 |
| Ningchun 52 | 0.544 | 9 | 0.552 | 12 | 0.507 | 11 |
| Yongliang 15 | 0.523 | 11 | 0.615 | 5 | 0.588 | 5 |
| 1407 | 0.523 | 10 | 0.594 | 8 | 0.587 | 6 |
| Longchun 35 | 0.519 | 12 | 0.695 | 4 | 0.657 | 4 |
| 9396 | 0.510 | 13 | 0.525 | 15 | 0.500 | 12 |
| Lingxia 35 | 0.490 | 14 | 0.599 | 7 | 0.488 | 13 |
| 2038 | 0.476 | 15 | 0.431 | 19 | 0.000 | 27 |
| L623 | 0.461 | 16 | 0.405 | 22 | 0.388 | 18 |
| Longchun 30 | 0.454 | 17 | 0.613 | 6 | 0.578 | 7 |
| L622 | 0.452 | 18 | 0.416 | 21 | 0.398 | 16 |
| SM14 | 0.427 | 19 | 0.178 | 27 | 0.098 | 25 |
| Bamai 20 | 0.378 | 20 | 0.526 | 14 | 0.000 | 26 |
| 1538 | 0.361 | 21 | 0.329 | 24 | 0.304 | 20 |
| Linmai 33 | 0.351 | 22 | 0.589 | 10 | 0.578 | 9 |
| Bamai 19 | 0.318 | 23 | 0.589 | 9 | 0.578 | 8 |
| Longchun 41 | 0.317 | 24 | 0.330 | 23 | 0.180 | 24 |
| Dingxi 40 | 0.304 | 25 | 0.277 | 26 | 0.254 | 23 |
| Ningchun 15 | 0.244 | 26 | 0.304 | 25 | 0.300 | 21 |
| Dingxi 49 | 0.182 | 27 | 0.419 | 20 | 0.345 | 19 |
| Dingxi 48 | 0.092 | 28 | 0.138 | 28 | 0.000 | 28 |

Note: Spearman’s rank correlation coefficients: ρ = 0.63 (D vs. EW-MFV, *p* < 0.001) and ρ = 0.57 (D vs. GM, *p* < 0.001).
