# Supplementary material for: Drought adaptation in spring wheat seedlings relies on coordinated deep root architecture and cortical tissue allocation
Source: Front Plant Sci. 2026 Jun 8;17:1846481. doi: 10.3389/fpls.2026.1846481 (PMC13285027; doi:10.3389/fpls.2026.1846481)
Supplement: Supplementary Table S4 — Grain yield-based drought tolerance coefficients of 28 spring wheat cultivars under drought stress in 2025. [file Table4.docx]

**Table S4** Grain yield-based drought tolerance coefficients of 28 spring wheat cultivars under drought stress in 2025.

| Variety | Grain yield per plot (GYP) |
| --- | --- |
| Ruichun1 | 1.020 |
| 1538 | 0.659 |
| 2038 | 0.620 |
| Ningchun 11 | 0.707 |
| 9396 | 0.576 |
| Longchun 30 | 0.566 |
| Longchun 34 | 0.807 |
| Linmai 33 | 0.656 |
| Ningchun 16 | 0.823 |
| Ningchun 4 | 0.789 |
| SM14 | 0.748 |
| Ningchun 57 | 0.858 |
| Ningchun 15 | 0.533 |
| Ningchun 52 | 0.853 |
| Bamai 19 | 0.524 |
| Longchun 41 | 0.626 |
| L623 | 0.638 |
| L622 | 0.705 |
| Yongliang 15 | 0.674 |
| Dingxi 49 | 0.439 |
| Dingxi 48 | 0.405 |
| Dingxi 40 | 0.403 |
| Lingxia 35 | 0.758 |
| Bamai 20 | 0.717 |
| Longchun 35 | 0.527 |
| 1407 | 0.690 |
| Ningchun 32 | 0.823 |
| Yong 2563 | 0.578 |
| Mean | 0.669 |
| Max | 1.020 |
| Min | 0.403 |
| SD | 0.146 |
| CV(%) | 21.82 |
