# Supplementary figures and images for: Drought adaptation in spring wheat seedlings relies on coordinated deep root architecture and cortical tissue allocation
Source: Front Plant Sci. 2026 Jun 8;17:1846481. doi: 10.3389/fpls.2026.1846481 (PMC13285027; doi:10.3389/fpls.2026.1846481)

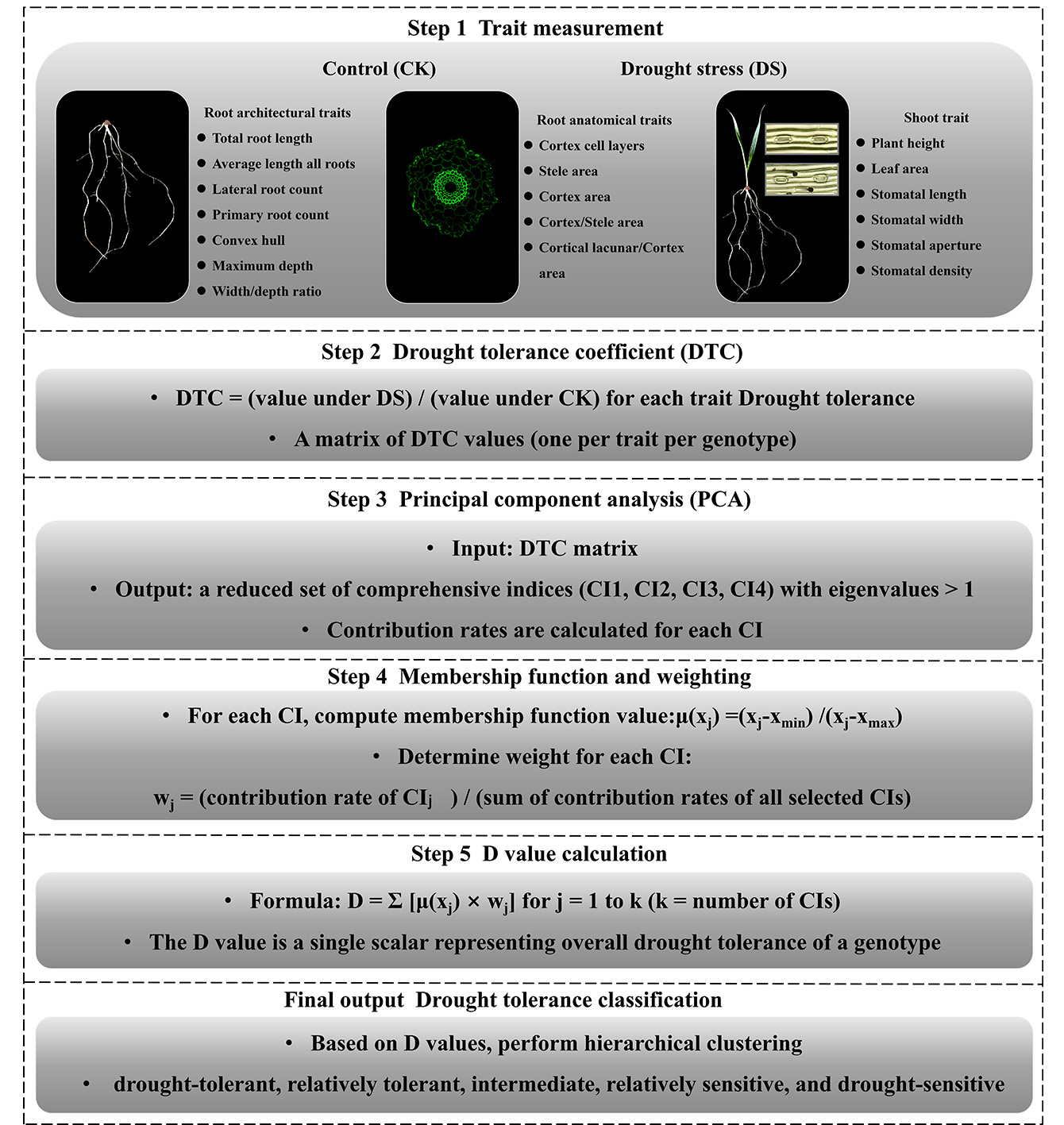

Supplement: Supplementary Figure S1 — Flowchart showing the calculation of the D value from root and shoot traits. Steps: (1) Trait measurement; (2) Drought tolerance coefficients; (3) PCA; (4) Membership function and weighting; (5) D value; (6) Drought tolerance classification. [file Image1.jpg]

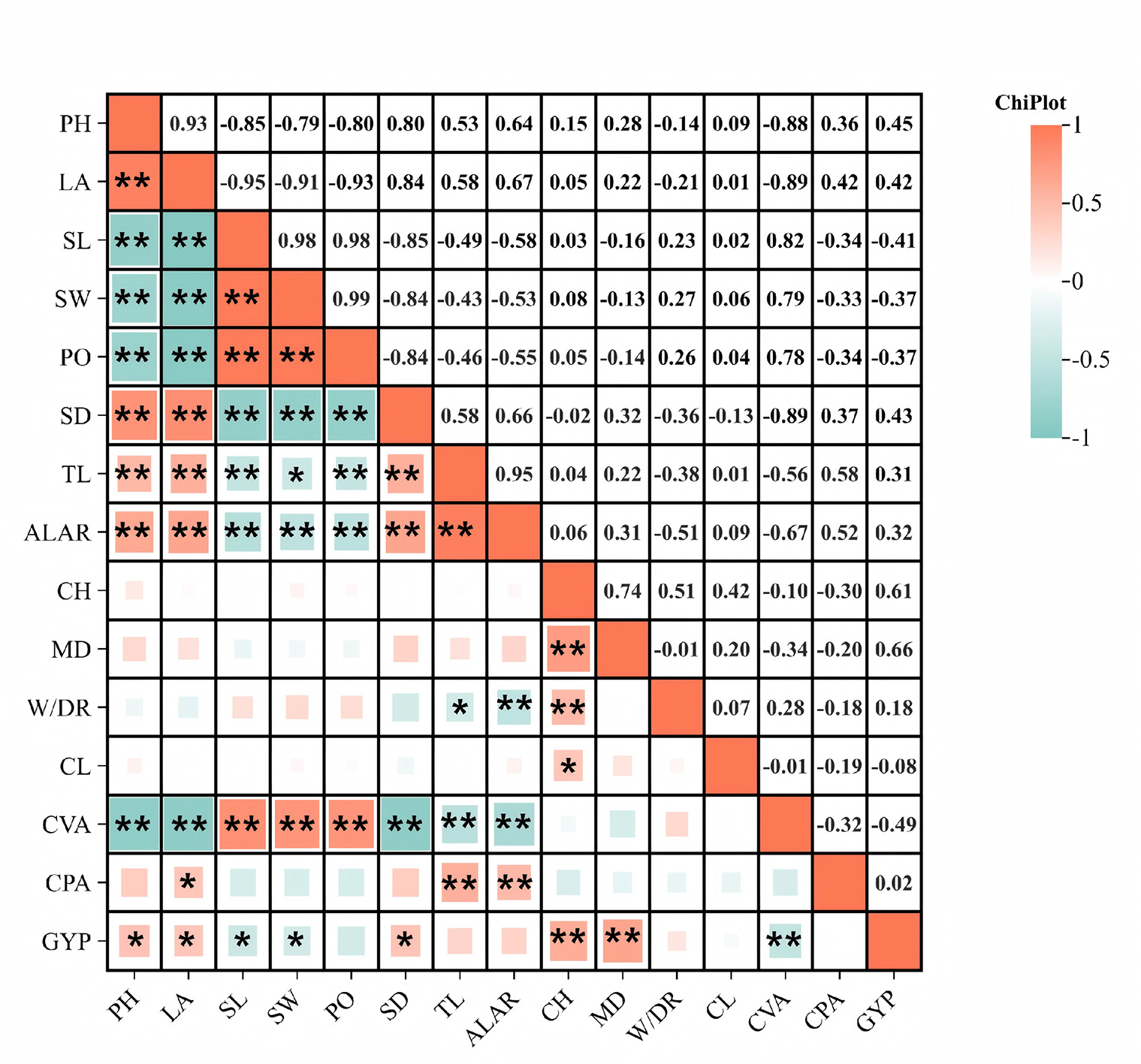

Supplement: Supplementary Figure S2 — Correlation analysis between plot grain yield and seedling stage drought stress traits. PH, Plant height; LA, Leaf area; SL, Stomatal length; SW, Stomatal width; PO, Stomatal aperture; SD, Stomatal density; TL, Total root length; ALAR, Total length of all roots; CH, Convex hull area; MD, Maximum root depth; W/DR, Width/depth ratio; CL, Number of cortical layers; CVA, Cortical/stele area; CPA, Aerenchyma/cortical area; GYP, Grain yield per plot. [file Image2.jpg]

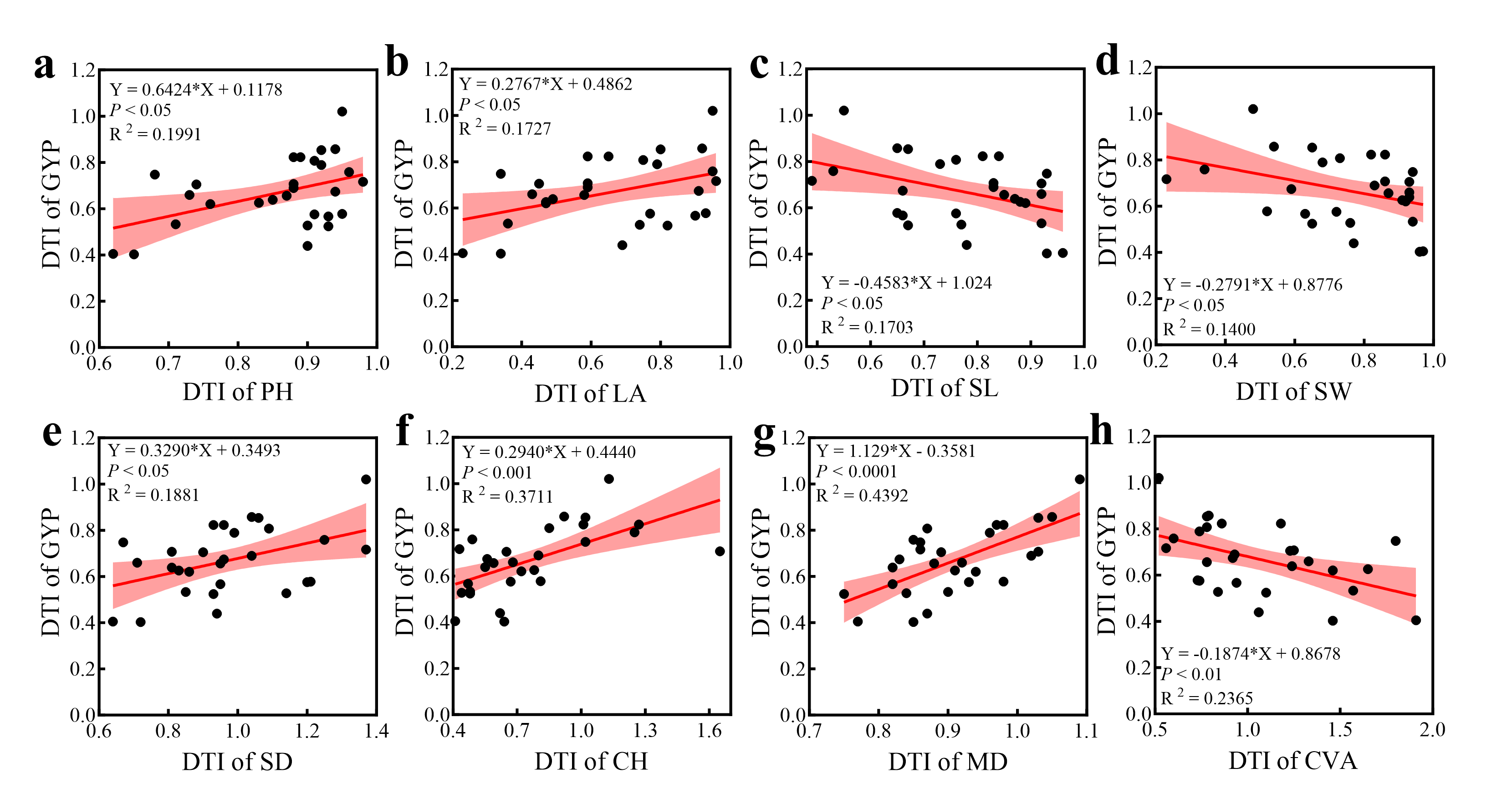

Supplement: Supplementary Figure S3 — Linear model fitting under drought stress, illustrating the relationship between the grain yield per plot drought tolerance index and the drought tolerance index of various seedling traits. PH, Plant height; LA, Leaf area; SL, Stomatal length; SW, Stomatal width; SD, Stomatal density; CH, Convex hull area; MD, Maximum root depth; CVA, Cortical/stele area; GYP, Grain yield per plot. [file Image3.tif]

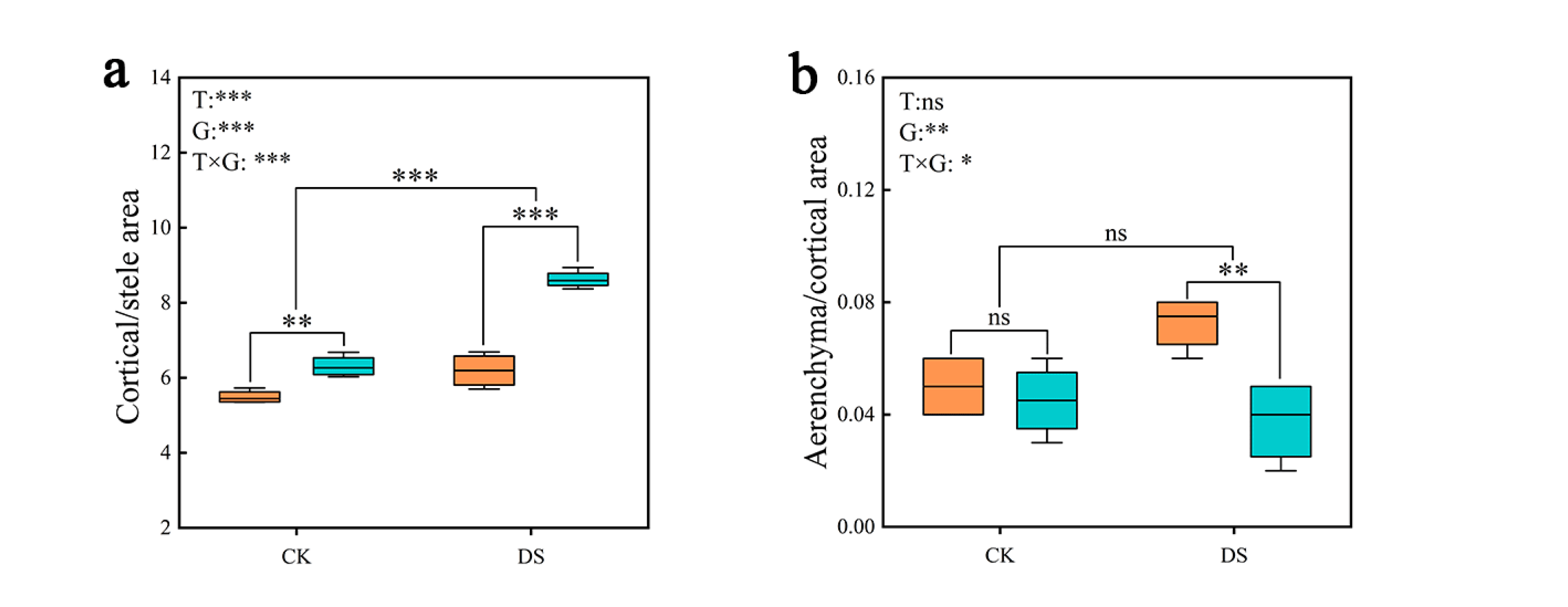

Supplement: Supplementary Figure S4 — Root anatomical characteristics of two spring wheat cultivars with significant responses in the 6–9 cm depth zone of growth pouches under drought stress. * p< 0.05; ** p< 0.01; *** p < 0.001; ns, Non-significant. [file Image4.tif]
